# Sequential linear regression with online standardized data

**DOI:** 10.1371/journal.pone.0191186

**Published:** 2018-01-18

**Authors:** Kévin Duarte, Jean-Marie Monnez, Eliane Albuisson

**Affiliations:** 1 Université de Lorraine, Institut Elie Cartan de Lorraine, UMR 7502, Vandoeuvre-lès-Nancy, F-54506, France; 2 Project-Team BIGS, INRIA, Villers-lès-Nancy, F-54600, France; 3 INSERM U1116, Centre d’Investigations Cliniques-Plurithématique 1433, Université de Lorraine, Nancy, France; 4 Université de Lorraine, Institut Universitaire de Technologie Nancy-Charlemagne, Nancy, F-54052, France; 5 BIOBASE, Pôle S²R, CHRU de Nancy, Vandoeuvre-lès-Nancy, France; 6 Faculté de Médecine, InSciDenS, Vandoeuvre-lès-Nancy, France; National University of Defense Technology, CHINA

## Abstract

The present study addresses the problem of sequential least square multidimensional linear regression, particularly in the case of a data stream, using a stochastic approximation process. To avoid the phenomenon of numerical explosion which can be encountered and to reduce the computing time in order to take into account a maximum of arriving data, we propose using a process with online standardized data instead of raw data and the use of several observations per step or all observations until the current step. Herein, we define and study the almost sure convergence of three processes with online standardized data: a classical process with a variable step-size and use of a varying number of observations per step, an averaged process with a constant step-size and use of a varying number of observations per step, and a process with a variable or constant step-size and use of all observations until the current step. Their convergence is obtained under more general assumptions than classical ones. These processes are compared to classical processes on 11 datasets for a fixed total number of observations used and thereafter for a fixed processing time. Analyses indicate that the third-defined process typically yields the best results.

## 1 Introduction

In the present analysis, *A*′ denotes the transposed matrix of *A* while the abbreviation “a.s.” signifies almost surely.

Let *R* = (*R*^1^,…,*R*^*p*^) and *S* = (*S*^1^,…,*S*^*q*^) be random vectors in $R^{p}$ and $R^{q}$ respectively. Considering the least square multidimensional linear regression of *S* with respect to *R*: the (*p*, *q*) matrix *θ* and the (*q*, 1) matrix *η* are estimated such that *E*[‖*S* − *θ*′ *R* − *η*‖^2^] is minimal.

Denote the covariance matrices

$$\begin{array}{ccc} B & = & Covar[R]=E[(R-E[R]){\left(R-E[R]\right)}^{\prime }],\\ F & = & Covar[R,S]=E[(R-E[R]){\left(S-E[S]\right)}^{\prime }]. \end{array}$$

If we assume *B* is positive definite, i.e. there is no affine relation between the components of *R*, then

$$\begin{array}{c} \theta =B^{-1}F,\eta =E[S]-\theta^{\prime }E[R]. \end{array}$$

Note that, *R*_1_ denoting the random vector in $R^{p+1}$ such that $R_{1}^{\prime }=(\begin{array}{cc} R^{\prime } & 1 \end{array})$, *θ*_1_ the (*p* + 1, *q*) matrix such that $\theta_{1}^{\prime }=(\begin{array}{cc} \theta^{\prime } & \eta \end{array})$, $B_{1}=E[R_{1}R_{1}^{\prime }]$ and *F*_1_ = *E*[*R*_1_ *S*′], we obtain $\theta_{1}=B_{1}^{-1}F_{1}$.

In order to estimate *θ* (or *θ*_1_), a stochastic approximation process (*X*_*n*_) in $R^{p\times q}$ (or $R^{(p+1)\times q}$) is recursively defined such that

$$\begin{array}{c} X_{n+1}=X_{n}-a_{n}(B_{n}X_{n}-F_{n}), \end{array}$$

where (*a*_*n*_) is a sequence of positive real numbers, eventually constant, called step-sizes (or gains). Matrices *B*_*n*_ and *F*_*n*_ have the same dimensions as *B* and *F*, respectively. The convergence of (*X*_*n*_) towards *θ* is studied under appropriate definitions and assumptions on *B*_*n*_ and *F*_*n*_.

Suppose that ((*R*_1*n*_, *S*_*n*_), *n* ≥ 1) is an i.i.d. sample of (*R*_1_, *S*). In the case where *q* = 1, $B_{n}=R_{1n}R_{1n}^{\prime }$ and $F_{n}=R_{1n}S_{n}^{\prime }$, several studies have been devoted to this stochastic gradient process (see for example Monnez [1], Ljung [2] and references hereafter). In order to accelerate general stochastic approximation procedures, Polyak [3] and Polyak and Juditsky [4] introduced the averaging technique. In the case of linear regression, Györfi and Walk [5] studied an averaged stochastic approximation process with a constant step-size. With the same type of process, Bach and Moulines [6] proved that the optimal convergence rate is achieved without strong convexity assumption on the loss function.

However, this type of process may be subject to the risk of numerical explosion when components of *R* or *S* exhibit great variances and may have very high values. For datasets used as test sets by Bach and Moulines [6], all sample points whose norm of *R* is fivefold greater than the average norm are removed. Moreover, generally only one observation of (*R*, *S*) is introduced at each step of the process. This may be not convenient for a large amount of data generated by a data stream for example.

Two modifications of this type of process are thus proposed in this article.

The first change in order to avoid numerical explosion is the use of standardized, i.e. of zero mean and unit variance, components of *R* and *S*. In fact, the expectation and the variance of the components are usually unknown and will be estimated online.

The parameter *θ* can be computed from the standardized components as follows. Let *σ*^*j*^ the standard deviation of *R*^*j*^ for *j* = 1,…,*p* and $\sigma_{1}^{k}$ the standard deviation of *S*^*k*^ for *k* = 1,…,*q*. Define the following matrices

$$\begin{array}{c} \Gamma =(\begin{array}{ccc} \frac{1}{\sigma^{1}} & \cdots & 0\\ \vdots & \ddots & \vdots \\ 0 & \cdots & \frac{1}{\sigma^{p}} \end{array}),\Gamma^{1}=(\begin{array}{ccc} \frac{1}{\sigma_{1}^{1}} & \cdots & 0\\ \vdots & \ddots & \vdots \\ 0 & \cdots & \frac{1}{\sigma_{1}^{q}} \end{array}). \end{array}$$

Let *S*_*c*_ = Γ^1^(*S* − *E*[*S*]) and *R*_*c*_ = Γ(*R* − *E*[*R*]). The least square linear regression of *S*_*c*_ with respect to *R*_*c*_ is achieved by estimating the (*p*, *q*) matrix *θ*_*c*_ such that $E[{||S_{c}-\theta_{c}^{{}^{\prime }}R_{c}||}^{2}]$ is minimal. Then *θ*_*c*_ = Γ^−1^(*B*^−1^ *F*)Γ^1^ ⇔ *θ* = *B*^−1^ *F* = Γ*θ*_*c*_(Γ^1^)^−1^.

The second change is to use, at each step of the process, several observations of (*R*, *S*) or an estimation of *B* and *F* computed recursively from all observations until the current step without storing them.

More precisely, the convergence of three processes with online standardized data is studied in sections 2, 3, 4 respectively.

First, in section 2, a process with a variable step-size *a*_*n*_ and use of several online standardized observations at each step is studied; note that the number of observations at each step may vary with *n*.

Secondly, in section 3, an averaged process with a constant step-size and use of a varying number of online standardized observations at each step is studied.

Thirdly, in section 4, a process with a constant or variable step-size and use of all online standardized observations until the current step to estimate *B* and *F* is studied.

These three processes are tested on several datasets when *q* = 1, *S* being a continuous or binary variable, and compared to existing processes in section 5. Note that when *S* is a binary variable, linear regression is equivalent to a linear discriminant analysis. It appears that the third-defined process most often yields the best results for the same number of observations used or for the same duration of computing time used.

These processes belong to the family of stochastic gradient processes and are adapted to data streams. Batch gradient and stochastic gradient methods are presented and compared in [7] and reviewed in [8], including noise reduction methods, like dynamic sample sizes methods, stochastic variance reduced gradient (also studied in [9]), second-order methods, ADAGRAD [10] and other methods. This work makes the following contributions to the variance reduction methods:

- In [9], the authors proposed a modification of the classical stochastic gradient algorithm to reduce directly the gradient of the function to be optimized in order to obtain a faster convergence. It is proposed in this article to reduce this gradient by an online standardization of the data.
- Gradient clipping [11] is another method to avoid a numerical explosion. The idea is to limit the norm of the gradient to a maximum number called threshold. This number must be chosen, a bad choice of threshold can affect the computing speed. Moreover it is then necessary to compare the norm of the gradient to this threshold at each step. In our approach the limitation of the gradient is implicitly obtained by online standardization of the data.
- If the expectation and the variance of the components of R and S were known, standardization of these variables could be made directly and convergence of the processes obtained using existing theorems. But these moments are unknown in the case of a data stream and are estimated online in this study. Thus the assumptions of the theorems of almost sure (a.s.) convergence of the processes studied in sections 2 and 3 and the corresponding proofs are more general than the classical ones in the linear regression case [1–5].
- The process defined in section 4 is not a classical batch method. Indeed in this type of method (gradient descent), the whole set of data is known a priori and is used at each step of the process. In the present study, new data are supposed to arrive at each step, as in a data stream, and are added to the preceding set of data, thus reducing by averaging the variance. This process can be considered as a dynamic batch method.
- A suitable choice of step-size is often crucial for obtaining good performance of a stochastic gradient process. If the step-size is too small, the convergence will be slower. Conversely, if the step-size is too large, a numerical explosion may occur during the first iterations. Following [6], a very simple choice of the step-size is proposed for the methods with a constant step-size.
- Another objective is to reduce computing time in order to take into account a maximum of data in the case of a data stream. It appears in the experiments that the use of all observations until the current step without storing them, several observations being introduced at each step, increases at best in general the convergence speed of the process. Moreover this can reduce the influence of outliers.

As a whole the major contributions of this work are to reduce gradient variance by online standardization of the data or use of a “dynamic” batch process, to avoid numerical explosions, to reduce computing time and consequently to better adapt the stochastic approximation processes used to the case of a data stream.

## 2 Convergence of a process with a variable step-size

Let (*B*_*n*_, *n* ≥ 1) and (*F*_*n*_, *n* ≥ 1) be two sequences of random matrices in $R^{p\times p}$ and $R^{p\times q}$ respectively. In this section, the convergence of the process (*X*_*n*_, *n* ≥ 1) in $R^{p\times q}$ recursively defined by

$$\begin{array}{c} X_{n+1}=X_{n}-a_{n}(B_{n}X_{n}-F_{n}) \end{array}$$

and its application to sequential linear regression are studied.

### 2.1 Theorem

Let *X*_1_ be a random variable in $R^{p\times q}$ independent from the sequence of random variables ((*B*_*n*_, *F*_*n*_), *n* ≥ 1) in $R^{p\times p}\times R^{p\times q}$.

Denote *T*_*n*_ the *σ*-field generated by *X*_1_ and (*B*_1_, *F*_1_),…,(*B*_*n*−1_, *F*_*n*−1_). *X*_1_, *X*_2_,…,*X*_*n*_ are *T*_*n*_-measurable.

Let (*a*_*n*_) be a sequence of positive numbers.

Make the following assumptions:

(H1a) There exists a positive definite symmetrical matrix *B* such that a.s.

1) $\sum_{n=1}^{\infty }a_{n}||E[B_{n}|T_{n}]-B||<\infty$

2) $\sum_{n=1}^{\infty }a_{n}^{2}E[{||B_{n}-B||}^{2}|T_{n}]<\infty$.

(H2a) There exists a matrix *F* such that a.s.

1)$\sum_{n=1}^{\infty }a_{n}||E[F_{n}|T_{n}]-F||<\infty$

2) $\sum_{n=1}^{\infty }a_{n}^{2}E[{||F_{n}-F||}^{2}|T_{n}]<\infty$.

(H3a) $\sum_{n=1}^{\infty }a_{n}=\infty ,\sum_{n=1}^{\infty }a_{n}^{2}<\infty$.

**Theorem 1** *Suppose H1a, H2a and H3a hold*. *Then X*_*n*_ *converges to θ* = *B*^−1^ *F a.s*.

State the Robbins-Siegmund lemma [12] used in the proof.

**Lemma 2** *Let* (Ω, *A*, *P*) *be a probability space and* (*T*_*n*_) *a non-decreasing sequence of sub*-*σ*-*fields of A*. *Suppose for all n*, *z*_*n*_, *α*_*n*_, *β*_*n*_ *and γ*_*n*_ *are four integrable non-negative T*_*n*_-*measurable random variables defined on* (Ω, *A*, *P*) *such that*:

$$\begin{array}{ccc} E[z_{n+1}|T_{n}] & \leq z_{n}(1+\alpha_{n})+\beta_{n}-\gamma_{n} & a.s. \end{array}$$

*Then, in the set* $\left\{\sum_{n=1}^{\infty }\alpha_{n}<\infty ,\sum_{n=1}^{\infty }\beta_{n}<\infty \right\}$, (*z*_*n*_) *converges to a finite random variable and* $\sum_{n=1}^{\infty }\gamma_{n}<\infty$ *a.s*.

*Proof of Theorem 1*. The Frobenius norm ‖*A*‖ for a matrix *A* is used. Recall that, if ‖*A*‖_2_ denotes the spectral norm of *A*, ‖*AB*‖ ≤ ‖*A*‖_2_‖*B*‖.

$$\begin{array}{ccc} X_{n+1}-\theta & = & X_{n}-\theta -a_{n}(B_{n}X_{n}-F_{n})\\ & = & \left(I-a_{n}B)(X_{n}-\theta )-a_{n}((B_{n}-B)X_{n}-(F_{n}-F)\right) \end{array}$$

Denote *Z*_*n*_ = (*B*_*n*_ − *B*)*X*_*n*_ − (*F*_*n*_ − *F*) = (*B*_*n*_ − *B*)(*X*_*n*_ − *θ*) + (*B*_*n*_ − *B*)*θ* − (*F*_*n*_ − *F*) and $X_{n}^{1}=X_{n}-\theta$. Then:

$$\begin{array}{ccc} X_{n+1}^{1} & = & (I-a_{n}B)X_{n}^{1}-a_{n}Z_{n}\\ {||X_{n+1}^{1}||}^{2} & = & {||(I-a_{n}B)X_{n}^{1}||}^{2}-2a_{n}\left\langle (I-a_{n}B)X_{n}^{1},Z_{n}\right\rangle +a_{n}^{2}{||Z_{n}||}^{2}. \end{array}$$

Denote λ the smallest eigenvalue of B. As *a*_*n*_ → 0, we have for *n* sufficiently large

$$\begin{array}{c} ||I-a_{n}{B||}_{2}=1-a_{n}\lambda <1. \end{array}$$

Then, taking the conditional expectation with respect to *T*_*n*_ yields almost surely:

$$\begin{array}{ccc} E[{||X_{n+1}^{1}||}^{2}|T_{n}] & \leq & {\left(1-a_{n}\lambda \right)}^{2}{||X_{n}^{1}||}^{2}+2a_{n}\left|\left\langle (I-a_{n}B)X_{n}^{1},E[Z_{n}|T_{n}]\right\rangle \right|+\\ & & a_{n}^{2}E[{||Z_{n}||}^{2}|T_{n}],\\ E[Z_{n}|T_{n}] & = & (E[B_{n}|T_{n}]-B)X_{n}^{1}+(E[B_{n}|T_{n}]-B)\theta -(E[F_{n}|T_{n}]-F). \end{array}$$

Denoting

$$\begin{array}{ccc} \beta_{n} & = & ||E[B_{n}|T_{n}]-B||,\delta_{n}=||E[F_{n}|T_{n}]-F||,\\ b_{n} & = & E[{||B_{n}-B||}^{2}|T_{n}],d_{n}=E[{||F_{n}-F||}^{2}|T_{n}], \end{array}$$

we obtain, as $||X_{n}^{1}||\leq 1+{||X_{n}^{1}||}^{2}$:

$$\begin{array}{ccc} \left|\langle (I-a_{n}B)X_{n}^{1},E[Z_{n}|T_{n}]\rangle \right| & \leq & ||X_{n}^{1}||||E[Z_{n}|T_{n}]||\\ & \leq & {||X_{n}^{1}||}^{2}(\beta_{n}(1+||\theta ||)+\delta_{n})+\beta_{n}||\theta ||+\delta_{n},\\ E[{||Z_{n}||}^{2}|T_{n}] & \leq & 3b_{n}{||X_{n}^{1}||}^{2}+3b_{n}{||\theta ||}^{2}+3d_{n},\\ E[{||X_{n+1}^{1}||}^{2}|T_{n}] & \leq & (1+a_{n}^{2}\lambda^{2}+2(1+||\theta ||)a_{n}\beta_{n}+2a_{n}\delta_{n}+3a_{n}^{2}b_{n}){||X_{n}^{1}||}^{2}+\\ & & 2||\theta ||a_{n}\beta_{n}+2a_{n}\delta_{n}+3{||\theta ||}^{2}a_{n}^{2}b_{n}+3a_{n}^{2}d_{n}-2a_{n}\lambda {||X_{n}^{1}||}^{2}. \end{array}$$

Applying Robbins-Siegmund lemma under assumptions H1a, H2a and H3a implies that there exists a non-negative random variable *T* such that a.s.

$$\begin{array}{c} ||X_{n}^{1}||⟶T,\sum_{n=1}^{\infty }a_{n}{||X_{n}^{1}||}^{2}<\infty . \end{array}$$

As $\sum_{n=1}^{\infty }a_{n}=\infty$, *T* = 0 a.s. ∎

A particular case with the following assumptions is now studied.

(H1a’) There exist a positive definite symmetrical matrix *B* and a positive real number *b* such that a.s.

1) for all *n*, *E*[*B*_*n*_|*T*_*n*_] = *B*

2) $\begin{array}{c} \sup\\ n \end{array}$ *E*[‖*B*_*n*_ − *B*‖^2^|*T*_*n*_] < *b*.

(H2a’) There exist a matrix *F* and a positive real number *d* such that a.s.

1) for all *n*, *E*[*F*_*n*_|*T*_*n*_] = *F*

2) $\begin{array}{c} \sup\\ n \end{array}$ *E*[‖*F*_*n*_ − *F*‖^2^|*T*_*n*_] < *d*.

(H3a’) Denoting λ the smallest eigenvalue of *B*,

$\left(a_{n}=\frac{a}{n^{\alpha }},a>0,\frac{1}{2}<\alpha <1\right)$ or $\left(a_{n}=\frac{a}{n},a>\frac{1}{2\lambda }\right)$.

**Theorem 3** *Suppose H1a’, H2a’ and H3a’ hold*. *Then X*_*n*_ *converges to θ almost surely and in quadratic mean*. *Moreover* $\bar{\lim}\frac{1}{a_{n}}E[{||X_{n}-\theta ||}^{2}]<\infty$.

*Proof of Theorem 3*. In the proof of theorem 1, take *β*_*n*_ = 0, *δ*_*n*_ = 0, *b*_*n*_ < *b*, *d*_*n*_ < *d*; then a.s.:

$$\begin{array}{c} E[{||X_{n+1}^{1}||}^{2}|T_{n}]\leq (1+\lambda^{2}a_{n}^{2}+3ba_{n}^{2}){||X_{n}^{1}||}^{2}+3(b{||\theta ||}^{2}+d)a_{n}^{2}-2a_{n}\lambda {||X_{n}^{1}||}^{2}. \end{array}$$

Taking the mathematical expectation yields:

$$\begin{array}{c} E[{||X_{n+1}^{1}||}^{2}]\leq (1+(\lambda^{2}+3b)a_{n}^{2})E[{||X_{n}^{1}||}^{2}]+3(b{||\theta ||}^{2}+d)a_{n}^{2}-2a_{n}\lambda E[{||X_{n}^{1}||}^{2}]. \end{array}$$

By Robbins-Siegmund lemma:

$$\begin{array}{c} \exists t\geq 0:E[{||X_{n}^{1}||}^{2}]⟶t;\sum_{n=1}^{\infty }a_{n}E[{||X_{n}^{1}||}^{2}]<\infty . \end{array}$$

As $\sum_{n=1}^{\infty }a_{n}=\infty$, *t* = 0. Therefore, there exist N∈N and *f* > 0 such that for *n* > *N*:

$$\begin{array}{c} E[{||X_{n+1}^{1}||}^{2}]\leq (1-2a_{n}\lambda )E[{||X_{n}^{1}||}^{2}]+fa_{n}^{2}. \end{array}$$

Applying a lemma of Schmetterer [13] for $a_{n}=\frac{a}{n^{\alpha }}$ with $\frac{1}{2}<\alpha <1$ yields:

$$\begin{array}{c} \bar{\lim}n^{\alpha }E[{||X_{n}^{1}||}^{2}]<\infty . \end{array}$$

Applying a lemma of Venter [14] for $a_{n}=\frac{a}{n}$ with $a>\frac{1}{2\lambda }$ yields:

$$\begin{array}{c} \bar{\lim}nE[{||X_{n}^{1}||}^{2}]<\infty \;∎ \end{array}$$

### 2.2 Application to linear regression with online standardized data

Let (*R*_1_, *S*_1_),…,(*R*_*n*_, *S*_*n*_),… be an i.i.d. sample of a random vector (*R*, *S*) in $R^{p}\times R^{q}$. Let Γ (respectively Γ^1^) be the diagonal matrix of order *p* (respectively *q*) of the inverses of the standard deviations of the components of *R* (respectively *S*).

Define the correlation matrices

$$\begin{array}{c} B=\Gamma E[(R-E[R]){\left(R-E[R]\right)}^{\prime }]\Gamma ,\\ F=\Gamma E[(R-E[R]){\left(S-E[S]\right)}^{\prime }]\Gamma^{1}. \end{array}$$

Suppose that *B*^−1^ exists. Let *θ* = *B*^−1^ *F*.

Denote ${\bar{R}}_{n}$ (respectively ${\bar{S}}_{n}$) the mean of the *n*-sample (*R*_1_, *R*_2_,…,*R*_*n*_) of *R* (respectively (*S*_1_, *S*_2_,…,*S*_*n*_) of *S*).

Denote ${\left(V_{n}^{j}\right)}^{2}$ the variance of the *n*-sample $\left(R_{1}^{j},R_{2}^{j},...,R_{n}^{j}\right)$ of the *j*^*th*^ component *R*^*j*^ of *R*, and ${\left(V_{n}^{1k}\right)}^{2}$ the variance of the *n*-sample $\left(S_{1}^{k},S_{2}^{k},...,S_{n}^{k}\right)$ of the *k*^*th*^ component *S*^*k*^ of *S*.

Denote Γ_*n*_ (respectively $\Gamma_{n}^{1}$) the diagonal matrix of order *p* (respectively *q*) whose element (*j*, *j*) (respectively (*k*, *k*)) is the inverse of $\sqrt{\frac{n}{n-1}}V_{n}^{j}$ (respectively $\sqrt{\frac{n}{n-1}}V_{n}^{1k}$).

Let (*m*_*n*_, *n* ≥ 1) be a sequence of integers. Denote $M_{n}=\sum_{k=1}^{n}m_{k}$ for *n* ≥ 1, *M*_0_ = 0 and *I*_*n*_ = {*M*_*n*−1_+1,…,*M*_*n*_}.

Define

$$\begin{array}{cc} B_{n} & =\Gamma_{M_{n-1}}\frac{1}{m_{n}}\sum_{j\in I_{n}}(R_{j}-{\bar{R}}_{M_{n-1}}){\left(R_{j}-{\bar{R}}_{M_{n-1}}\right)}^{\prime }\Gamma_{M_{n-1}},\\ F_{n} & =\Gamma_{M_{n-1}}\frac{1}{m_{n}}\sum_{j\in I_{n}}(R_{j}-{\bar{R}}_{M_{n-1}}){\left(S_{j}-{\bar{S}}_{M_{n-1}}\right)}^{\prime }\Gamma_{M_{n-1}}^{1}. \end{array}$$

Define recursively the process (*X*_*n*_, *n* ≥ 1) in $R^{p\times q}$ by

$$\begin{array}{c} X_{n+1}=X_{n}-a_{n}(B_{n}X_{n}-F_{n}). \end{array}$$

**Corollary 4** *Suppose there is no affine relation between the components of R and the moments of order 4 of* (*R*, *S*) *exist*. *Suppose moreover that assumption H3a” holds*:

(*H3a*”) $a_{n}>0,\sum_{n=1}^{\infty }\frac{a_{n}}{\sqrt{n}}<\infty ,\sum_{n=1}^{\infty }a_{n}^{2}<\infty .$

*Then X*_*n*_ *converges to θ a.s*.

This process was tested on several datasets and some results are given in section 5 (process S11 for *m*_*n*_ = 1 and S12 for *m*_*n*_ = 10).

The following lemma is first proved.

**Lemma 5** *Suppose the moments of order 4 of R exist and a*_*n*_ > 0, $\sum_{n=1}^{\infty }\frac{a_{n}}{\sqrt{n}}<\infty$. *Then* $\sum_{n=1}^{\infty }a_{n}||{\bar{R}}_{M_{n-1}}-E[R]||<\infty$ *and* $\sum_{n=1}^{\infty }a_{n}||\Gamma_{M_{n-1}}-\Gamma ||<\infty$ *a.s*.

*Proof of Lemma 5*. The usual Euclidean norm for vectors and the spectral norm for matrices are used in the proof.

Step 1:

Denote $Var[R]=E[{||R-E[R]||}^{2}]=\sum_{j=1}^{p}Var[R^{j}]$.

$$\begin{array}{ccc} E[{||{\bar{R}}_{M_{n-1}}-E[R]||}^{2}] & = & \sum_{j=1}^{p}Var[{\bar{R}}_{M_{n-1}}^{j}]=\sum_{j=1}^{p}\frac{Var[R^{j}]}{M_{n-1}}\leq \frac{Var[R]}{n-1}. \end{array}$$

Then:

$$\begin{array}{ccc} \sum_{n=1}^{\infty }a_{n}E[||{\bar{R}}_{M_{n-1}}-E[R]||] & \leq & \sqrt{Var[R]}\sum_{n=1}^{\infty }\frac{a_{n}}{\sqrt{n-1}}<\infty \;\text{by}\;\text{H}3\text{a}”. \end{array}$$

It follows that $\sum_{n=1}^{\infty }a_{n}||{\bar{R}}_{M_{n-1}}-E[R]||<\infty$ a.s.

Likewise $\sum_{n=1}^{\infty }a_{n}||{\bar{S}}_{M_{n-1}}-E[S]||<\infty$ a.s.

Step 2:

$$\begin{array}{ll} \left||\Gamma_{M_{n-1}}-\Gamma |\right| & =\underset{j=1,\ldots ,p}{\text{max}}\left|\frac{1}{\sqrt{\frac{M_{n-1}}{M_{n-1}-1}}V_{M_{n-1}}^{j}}-\frac{1}{\sqrt{Var\left[R^{j}\right]}}\right|\\ & \leq \sum_{j=1}^{p}\frac{\left|\sqrt{\frac{M_{n-1}}{M_{n-1}-1}}V_{M_{n-1}}^{j}-\sqrt{Var\left[R^{j}\right]}\right|}{\sqrt{\frac{M_{n-1}}{M_{n-1}-1}}V_{M_{n-1}}^{j}\sqrt{Var\left[R^{j}\right]}}\\ & =\sum_{j=1}^{p}\frac{\left|\frac{M_{n-1}}{M_{n-1}-1}{\left(V_{M_{n-1}}^{j}\right)}^{2}-Var\left[R^{j}\right]\right|}{\sqrt{\frac{M_{n-1}}{M_{n-1}-1}}V_{M_{n-1}}^{j}\sqrt{Var\left[R^{j}\right]}\left(\sqrt{\frac{M_{n-1}}{M_{n-1}-1}}V_{M_{n-1}}^{j}+\sqrt{Var\left[R^{j}\right]}\right)}. \end{array}$$

Denote $\mu_{4}^{j}$ the centered moment of order 4 of *R*^*j*^. We have:

$$\begin{array}{ccc} E[|\frac{M_{n-1}}{M_{n-1}-1}{\left(V_{M_{n-1}}^{j}\right)}^{2}-Var[R^{j}]|] & \leq & \sqrt{Var[\frac{M_{n-1}}{M_{n-1}-1}{\left(V_{M_{n-1}}^{j}\right)}^{2}]}\\ & = & O(\sqrt{\frac{\mu_{4}^{j}-{\left(Var[R^{j}]\right)}^{2}}{M_{n-1}}}). \end{array}$$

Then by H3a”, as *M*_*n*−1_ ≥ *n*−1:

$$\begin{array}{c} \sum_{n=1}^{\infty }a_{n}\sum_{j=1}^{p}E[|\frac{M_{n-1}}{M_{n-1}-1}{\left(V_{M_{n-1}}^{j}\right)}^{2}-Var[R^{j}]|]<\infty \\ \Rightarrow \sum_{n=1}^{\infty }a_{n}\sum_{j=1}^{p}|\frac{M_{n-1}}{M_{n-1}-1}{\left(V_{M_{n-1}}^{j}\right)}^{2}-Var\left[R^{j}\right]|<\infty \;\text{a}.\text{s}. \end{array}$$

As ${\left(V_{M_{n-1}}^{j}\right)}^{2}\to Var[R^{j}]$ a.s., *j* = 1,…,*p*, this implies:

$$\begin{array}{c} \sum_{n=1}^{\infty }a_{n}||\Gamma_{M_{n-1}}-\Gamma ||<\infty \;\text{a}.\text{s}.∎ \end{array}$$

*Proof of Corollary 4*.

Step 1: prove that assumption H1a1 of theorem 1 is verified.

Denote *R*^*c*^ = *R* − *E*[*R*], $R_{j}^{c}=R_{j}-E[R]$, ${\bar{R}}_{j}^{c}={\bar{R}}_{j}-E[R]$.

$$\begin{array}{ccc} B_{n} & = & \Gamma_{M_{n-1}}\frac{1}{m_{n}}\sum_{j\in I_{n}}(R_{j}^{c}-{\bar{R}}_{M_{n-1}}^{c}){\left(R_{j}^{c}-{\bar{R}}_{M_{n-1}}^{c}\right)}^{\prime }\Gamma_{M_{n-1}}\\ & = & \Gamma_{M_{n-1}}\frac{1}{m_{n}}\sum_{j\in I_{n}}(R_{j}^{c}{R_{j}^{c}}^{\prime }-{\bar{R}}_{M_{n-1}}^{c}{R_{j}^{c}}^{\prime }-R_{j}^{c}{\left({\bar{R}}_{M_{n-1}}^{c}\right)}^{\prime }+{\bar{R}}_{M_{n-1}}^{c}{\left({\bar{R}}_{M_{n-1}}^{c}\right)}^{\prime })\Gamma_{M_{n-1}}.\\ B & = & \Gamma E[R^{c}{R^{c}}^{\prime }]\Gamma . \end{array}$$

As Γ_*M*_*n*−1__ and ${\bar{R}}_{M_{n-1}}$ are *T*_*n*_-measurable and $R_{j}^{c}$, *j* ∈ *I*_*n*_, is independent of *T*_*n*_, with $E[R_{j}^{c}]=0$:

$$\begin{array}{ccc} E[B_{n}|T_{n}]-B & = & \Gamma_{M_{n-1}}(E[R^{c}{R^{c}}^{\prime }]+{\bar{R}}_{M_{n-1}}^{c}{\left({\bar{R}}_{M_{n-1}}^{c}\right)}^{\prime })\Gamma_{M_{n-1}}-\Gamma E[R^{c}{R^{c}}^{\prime }]\Gamma \\ & = & \left(\Gamma_{M_{n-1}}-\Gamma )E[R^{c}{R^{c}}^{\prime }]\Gamma_{M_{n-1}}+\Gamma E[R^{c}{R^{c}}^{\prime }](\Gamma_{M_{n-1}}-\Gamma \right)\\ & & +\Gamma_{M_{n-1}}{\bar{R}}_{M_{n-1}}^{c}{\left({\bar{R}}_{M_{n-1}}^{c}\right)}^{\prime }\Gamma_{M_{n-1}}\;\text{a}.\text{s}. \end{array}$$

As Γ_*M*_*n*−1__ and ${\bar{R}}_{M_{n-1}}^{c}$ converge respectively to Γ and 0 a.s. and by lemma 5, $\sum_{n=1}^{\infty }a_{n}||\Gamma_{M_{n-1}}-\Gamma ||<\infty$ and $\sum_{n=1}^{\infty }a_{n}||{\bar{R}}_{M_{n-1}}^{c}||<\infty$ a.s., it follows that $\sum_{n=1}^{\infty }a_{n}||E[B_{n}|T_{n}]-B||<\infty$ a.s.

Step 2: prove that assumption H1a2 of theorem 1 is verified.

$$\begin{array}{ll} {\left\|B_{n}-B\right\|}^{2} & \leq 2{\left\|\Gamma_{M_{n-1}}\frac{1}{m_{n}}\sum_{j\in I_{n}}\left(R_{j}^{c}-{\bar{R}}_{M_{n-1}}^{c}\right){\left(R_{j}^{c}-{\bar{R}}_{M_{n-1}}^{c}\right)}^{\prime }\Gamma_{M_{n-1}}\right\|}^{2}\\ & +2{\left\|\Gamma E\left[R^{c}R^{c}{}^{\prime }\right]\Gamma \right\|}^{2}\\ & \leq 2{\left\|\Gamma_{M_{n-1}}\right\|}^{4}\frac{1}{m_{n}}\sum_{j\in I_{n}}{\left\|R_{j}^{c}-{\bar{R}}_{M_{n-1}}^{c}\right\|}^{4}+2{\left\|\Gamma E\left[R^{c}R^{c}{}^{\prime }\right]\Gamma \right\|}^{2}\\ & \leq 2{\left\|\Gamma_{M_{n-1}}\right\|}^{4}\frac{1}{m_{n}}\sum_{j\in I_{n}}2^{3}\left({\left\|R_{j}^{c}\right\|}^{4}+{\left\|{\bar{R}}_{M_{n-1}}^{c}\right\|}^{4}\right)+2{\left\|\Gamma E\left[R^{c}R^{c}{}^{\prime }\right]\Gamma \right\|}^{2}. \end{array}$$

$$\begin{array}{ccc} E[{||B_{n}-B||}^{2}|T_{n}] & \leq & 2^{4}{||\Gamma_{M_{n-1}}||}^{4}(E[{||R^{c}||}^{4}]+{||{\bar{R}}_{M_{n-1}}^{c}||}^{4})+2{||\Gamma E[R^{c}{R^{c}}^{\prime }]\Gamma ||}^{2}\;\text{a}.\text{s}. \end{array}$$

As Γ_*M*_*n*−1__ and ${\bar{R}}_{M_{n-1}}^{c}$ converge respectively to Γ and 0 a.s., and $\sum_{n=1}^{\infty }a_{n}^{2}<\infty$, it follows that $\sum_{n=1}^{\infty }a_{n}^{2}E[{||B_{n}-B||}^{2}|T_{n}]<\infty$ a.s.

Step 3: the proofs of the verification of assumptions H2a1 and H2a2 of theorem 1 are similar to the previous ones, *B*_*n*_ and *B* being respectively replaced by

$$\begin{array}{ccc} F_{n} & = & \Gamma_{M_{n-1}}\frac{1}{m_{n}}\sum_{j\in I_{n}}(R_{j}^{c}-{\bar{R}}_{M_{n-1}}^{c}){\left(S_{j}^{c}-{\bar{S}}_{M_{n-1}}^{c}\right)}^{\prime }\Gamma_{M_{n-1}}^{1},\\ F & = & \Gamma E[R^{c}{S^{c}}^{\prime }]\Gamma^{1}∎ \end{array}$$

## 3 Convergence of an averaged process with a constant step-size

In this section, the process (*X*_*n*_, *n* ≥ 1) with a constant step-size *a* and the averaged process (*Y*_*n*_, *n* ≥ 1) in $R^{p\times q}$ are recursively defined by

$$\begin{array}{ccc} X_{n+1} & = & X_{n}-a(B_{n}X_{n}-F_{n})\\ Y_{n+1} & = & \frac{1}{n+1}\sum_{j=1}^{n+1}X_{j}=Y_{n}-\frac{1}{n+1}(Y_{n}-X_{n+1}). \end{array}$$

The a.s. convergence of (*Y*_*n*_, *n* ≥ 1) and its application to sequential linear regression are studied.

### 3.1 Lemma

**Lemma 6** *Let three real sequences* (*u*_*n*_), (*v*_*n*_) *and* (*a*_*n*_), *with u*_*n*_ > 0 *and a*_*n*_ > 0 *for all n*, *and a real positive number λ such that, for n* ≥ 1,

$$\begin{array}{ccc} u_{n+1} & \leq & (1-a_{n}\lambda )u_{n}+a_{n}v_{n}. \end{array}$$

*Suppose*:

*1) v*_*n*_ → 0
*2)* $\left(a_{n}=a<\frac{1}{\lambda }\right)$ *or* $\left(a_{n}\to 0\text{,}\;\sum_{n=1}^{\infty }a_{n}=\infty \right)$.
*Under assumptions 1 and 2, u*_*n*_ → 0.

*Proof of Lemma 6*. In the case *a*_*n*_ depending on *n*, as *a*_*n*_ → 0, we can suppose without loss of generality that 1 − *a*_*n*_ λ > 0 for *n* ≥ 1. We have:

$$\begin{array}{ccc} u_{n+1} & \leq & \prod_{i=1}^{n}(1-a_{i}\lambda )u_{1}+\sum_{i=1}^{n}a_{i}\prod_{l=i+1}^{n}(1-a_{l}\lambda )v_{i}\text{,}\;\text{with}\prod_{n+1}^{n}=1. \end{array}$$

Now, for *n*_1_ ≤ *n*_2_ ≤ *n* and 0 < *c*_*i*_ < 1 with *c*_*i*_ = *a*_*i*_ *λ* for all *i*, we have:

$$\begin{array}{ccc} \sum_{i=n_{1}}^{n_{2}}c_{i}\prod_{l=i+1}^{n}(1-c_{l}) & = & \sum_{i=n_{1}}^{n_{2}}(1-(1-c_{i}))\prod_{l=i+1}^{n}(1-c_{l})\\ & = & \sum_{i=n_{1}}^{n_{2}}(\prod_{l=i+1}^{n}(1-c_{l})-\prod_{l=i}^{n}(1-c_{l}))\\ & = & \prod_{l=n_{2}+1}^{n}(1-c_{l})-\prod_{l=n_{1}}^{n}(1-c_{l})\leq \prod_{l=n_{2}+1}^{n}(1-c_{l})\leq 1. \end{array}$$

Let *ϵ* > 0. There exists *N* such that for *i* > *N*, $|v_{i}|<\frac{\epsilon }{3}\lambda$. Then for *n* ≥ *N*, applying the previous inequality with *c*_*i*_ = *a*_*i*_ *λ*, *n*_1_ = 1, *n*_2_ = *N*, yields:

$$\begin{array}{ccc} u_{n+1} & \leq & \prod_{i=1}^{n}(1-a_{i}\lambda )u_{1}+\sum_{i=1}^{N}a_{i}\lambda \prod_{l=i+1}^{n}(1-a_{l}\lambda )\frac{\left|v_{i}\right|}{\lambda }+\frac{\epsilon }{3}\sum_{i=N+1}^{n}a_{i}\lambda \prod_{l=i+1}^{n}(1-a_{l}\lambda )\\ & \leq & \prod_{i=1}^{n}(1-a_{i}\lambda )u_{1}+\frac{1}{\lambda }\max_{1\leq i\leq N}|v_{i}|\prod_{l=N+1}^{n}(1-a_{l}\lambda )+\frac{\epsilon }{3}. \end{array}$$

In the case *a*_*n*_ depending on *n*, *ln*(1 − *a*_*i*_ λ) ∼ −*a*_*i*_ λ as *a*_*i*_ → 0(*i* → ∞); then, as $\sum_{n=1}^{\infty }a_{n}=\infty$, $\prod_{l=N+1}^{n}(1-a_{l}\lambda )\to 0(n\to \infty )$.

In the case *a*_*n*_ = *a*, $\prod_{l=N+1}^{n}(1-a\lambda )={\left(1-a\lambda \right)}^{n-N}\to 0(n\to \infty )$ as 0 < 1 − *a*λ < 1.

Thus there exists *N*_1_ such that *u*_*n*+1_ < *ϵ* for *n* > *N*_1_ ∎

### 3.2 Theorem

Make the following assumptions

(H1b) There exist a positive definite symmetrical matrix *B* in $R^{p\times p}$ and a positive real number *b* such that a.s.

1) lim_*n* → ∞_(*E*[*B*_*n*_|*T*_*n*_] − *B*) = 0

2) $\sum_{n=1}^{\infty }\frac{1}{n}{\left(E[{||E[B_{n}|T_{n}]-B||}^{2}]\right)}^{\frac{1}{2}}<\infty$

3) sup_*n*_ *E*[‖*B*_*n*_−*B*‖^2^|*T*_*n*_] ≤ *b*.

(H2b) There exist a matrix *F* in $R^{p\times q}$ and a positive real number *d* such that a.s.

1) lim_*n*→∞_(*E*[*F*_*n*_|*T*_*n*_] − *F*) = 0

2) sup_*n*_ *E* [‖*F*_*n*_ − *F*‖^2^|*T*_*n*_] ≤ *d*.

(H3b) *λ* and *λ*_*max*_ being respectively the smallest and the largest eigenvalue of *B*, $0<a<\min(\frac{1}{\lambda_{max}},\frac{2\lambda }{\lambda^{2}+b})$.

**Theorem 7** *Suppose H1b, H2b and H3b hold*. *Then Y*_*n*_ *converges to θ* = *B*^−1^ *F a.s*.

**Remark 1** *Györfi and Walk* [5] *proved that Y*_*n*_ *converges to θ a*.*s*. *and in quadratic mean under the assumptions E*[*B*_*n*_|*T*_*n*_] = *B*, *E*[*F*_*n*_|*T*_*n*_] = *F*, *H1b2 and H2b2. Theorem 7 is an extension of their a.s. convergence result when E*[*B*_*n*_|*T*_*n*_] → *B and E*[*F*_*n*_|*T*_*n*_] → *F a.s*.

**Remark 2** *Define* $R_{1}=(\begin{array}{c} R\\ 1 \end{array})$, $B=E[R_{1}R_{1}^{\prime }]$, *F* = *E*[*R*_1_ *S*′]. *If* ((*R*_1*n*_, *S*_*n*_), *n* ≥ 1) *is an i.i.d. sample of* (*R*_1_, *S*) *whose moments of order 4 exist, assumptions H1b and H2b are verified for* $B_{n}=R_{1n}R_{1n}^{\prime }$ *and* $F_{n}=R_{1n}S_{n}^{\prime }$ *as* $E[R_{1n}R_{1n}^{\prime }|T_{n}]=E[R_{1}R_{1}^{\prime }]=B$ *and* $E[R_{1n}S_{n}^{\prime }|T_{n}]=F$.

*Proof of Theorem 7*. Denote

$$\begin{array}{ccc} Z_{n} & = & (B_{n}-B)(X_{n}-\theta )+(B_{n}-B)\theta -(F_{n}-F),\\ X_{n}^{1} & = & X_{n}-\theta ,\\ Y_{n}^{1} & = & Y_{n}-\theta =\frac{1}{n}\sum_{j=1}^{n}X_{j}^{1}. \end{array}$$

Step 1: give a sufficient condition to have $Y_{n}^{1}\to 0$ a.s.

We have (cf. proof of theorem 1):

$$\begin{array}{ccc} X_{n+1}^{1} & = & (I-aB)X_{n}^{1}-aZ_{n},\\ Y_{n+1}^{1} & = & \frac{1}{n+1}X_{1}^{1}+\frac{1}{n+1}\sum_{j=2}^{n+1}X_{j}^{1}\\ & = & \frac{1}{n+1}X_{1}^{1}+\frac{1}{n+1}\sum_{j=2}^{n+1}(I-aB)X_{j-1}^{1}-a\frac{1}{n+1}\sum_{j=2}^{n+1}Z_{j-1}\\ & = & \frac{1}{n+1}X_{1}^{1}+\frac{n}{n+1}(I-aB)Y_{n}^{1}-a\frac{1}{n+1}\sum_{j=1}^{n}Z_{j}. \end{array}$$

Take now the Frobenius norm of $Y_{n+1}^{1}$:

$$\begin{array}{ccc} \left\|Y_{n+1}^{1}\right\| & \leq & \|\left(I-aB\right)Y_{n}^{1}\|+a\|\frac{1}{n+1}\sum_{j=1}^{n}Z_{j}-\frac{1}{n+1}\frac{1}{a}X_{1}^{1}\|. \end{array}$$

Under H3b, all the eigenvalues of *I* − *aB* are positive and the spectral norm of *I* − *aB* is equal to 1 − *aλ*. Then:

$$\begin{array}{ccc} \left||Y_{n+1}^{1}|\right| & \leq & \left(1-a\lambda \right)\left\|Y_{n}^{1}\right\|+a\left\|\frac{1}{n+1}\sum_{j=1}^{n}Z_{j}-\frac{1}{n+1}\frac{1}{a}X_{1}^{1}\right\|. \end{array}$$

By lemma 6, it suffices to prove $\frac{1}{n}\sum_{j=1}^{n}Z_{j}\to 0$ a.s. to conclude $Y_{n}^{1}\to 0$ a.s.

Step 2: prove that assumptions H1b and H2b imply respectively $\frac{1}{n}\sum_{j=1}^{n}B_{j}\to B$ and $\frac{1}{n}\sum_{j=1}^{n}F_{j}\to F$ a.s.

The proof is only given for (*B*_*n*_), the other one being similar.

Assumption H1b3 implies sup_*n*_ *E*[‖*B*_*n*_ − *B*‖^2^] < ∞. It follows that, for each element $B_{n}^{kl}$ and *B*^*kl*^ of *B*_*n*_ and *B* respectively, $\sum_{n=1}^{\infty }\frac{Var[B_{n}^{kl}-B^{kl}]}{n^{2}}<\infty$. Therefore:

$$\begin{array}{c} \frac{1}{n}\sum_{j=1}^{n}(B_{j}^{kl}-B^{kl}-E[B_{j}^{kl}-B^{kl}|T_{j}])\to 0\;\text{a}.\text{s}. \end{array}$$

As $E[B_{j}^{kl}-B^{kl}|T_{j}]\to 0$ a.s. by H1b1, we have for each (*k*, *l*)

$$\begin{array}{c} \frac{1}{n}\sum_{j=1}^{n}(B_{j}^{kl}-B^{kl})⟶0\;\text{a}.\text{s}. \end{array}$$

Then $\frac{1}{n}\sum_{j=1}^{n}(B_{j}-B)\to 0$ a.s.

Step 3: prove now that $\frac{1}{n}\sum_{j=1}^{n}(B_{j}-B)X_{j}^{1}\to 0$ a.s.

Denote *β*_*n*_ = ‖*E*[*B*_*n*_|*T*_*n*_] − *B*‖ and *γ*_*n*_ = ‖*E*[*F*_*n*_|*T*_*n*_] − *F*‖. *β*_*n*_ → 0 and *γ*_*n*_ → 0 a.s. under H1b1 and H2b1. Then: ∀*δ* > 0, ∀*ε* > 0, ∃*N*(*δ*, *ε*): ∀*n* ≥ *N*(*δ*, *ε*),

$$\begin{array}{c} P(\{sup_{j>n}(\beta_{j})\leq \delta \}⋂\{sup_{j>n}(\gamma_{j})\leq \delta \})>1-\varepsilon . \end{array}$$

As $a<\frac{2\lambda }{\lambda^{2}+b}$, choose *η* such that:

$$\begin{array}{c} 0<\eta <\frac{1}{b}(\frac{2\lambda }{a}-(\lambda^{2}+b))\Leftrightarrow \lambda >\frac{a}{2}(\lambda^{2}+b+\eta b). \end{array}$$

Choose *δ* such that

$$\begin{array}{c} 0<\delta <\frac{1}{\left(1-a\lambda )(||\theta ||+2\right)}(\lambda -\frac{a}{2}(\lambda^{2}+b+\eta b)). \end{array}$$

Let *ε* be fixed. Denote *N*_0_ = *N*(*δ*, *ε*) and, for *n* > *N*_0_,

$$\begin{array}{ccc} G_{n} & = & (\{\underset{N_{0}<j\leq n}{\sup}(\beta_{j})\leq \delta \}⋂\{\underset{N_{0}<j\leq n}{\sup}(\gamma_{j})\leq \delta \}),\\ G & = & (\{\underset{j>N_{0}}{\sup}(\beta_{j})\leq \delta \}⋂\{\underset{j>N_{0}}{\sup}(\gamma_{j})\leq \delta \})=\underset{n>N_{0}}{⋂}G_{n}. \end{array}$$

Remark that *G*_*n*_ is *T*_*n*_-measurable and, *I*_*G*_ denoting the indicator of *G*,

$$\begin{array}{c} G\subset G_{n+1}\subset G_{n}\Leftrightarrow I_{G}\leq I_{G_{n+1}}\leq I_{G_{n}}. \end{array}$$

Step 3a: prove that $\sup_{n}E[{||X_{n}^{1}||}^{2}I_{G_{n}}]<\infty$.

$$\begin{array}{ccc} {||X_{n+1}^{1}||}^{2}I_{G_{n+1}} & \leq & {||X_{n+1}^{1}||}^{2}I_{G_{n}}={||(I-aB)X_{n}^{1}I_{G_{n}}-aZ_{n}I_{G_{n}}||}^{2}\\ & \leq & {||(I-aB)X_{n}^{1}I_{G_{n}}||}^{2}-2a\langle (I-aB)X_{n}^{1}I_{G_{n}},Z_{n}I_{G_{n}}\rangle +a^{2}{||Z_{n}I_{G_{n}}||}^{2}. \end{array}$$

As the spectral norm ‖*I* − *aB*‖ = 1 − *a*λ, taking the conditional expectation with respect to *T*_*n*_ yields a.s.

$$\begin{array}{ccc} E[{||X_{n+1}^{1}||}^{2}I_{G_{n+1}}|T_{n}] & \leq & {\left(1-a\lambda \right)}^{2}{||X_{n}^{1}I_{G_{n}}||}^{2}-2a\langle (I-aB)X_{n}^{1}I_{G_{n}},E[Z_{n}|T_{n}]I_{G_{n}}\rangle \\ & & +a^{2}E[{||Z_{n}I_{G_{n}}||}^{2}|T_{n}]. \end{array}$$

Now:

$$\begin{array}{ccc} ||E[Z_{n}|T_{n}]I_{G_{n}}|| & = & ||(E[B_{n}|T_{n}]-B)X_{n}^{1}I_{G_{n}}+(E[B_{n}|T_{n}]-B)\theta I_{G_{n}}\\ & & -(E[F_{n}|T_{n}]-F)I_{G_{n}}||\\ & \leq & \delta ||X_{n}^{1}I_{G_{n}}||+\delta (||\theta ||+1)\\ E[{||Z_{n}I_{G_{n}}||}^{2}|T_{n}] & \leq & \left(1+\eta )E[{||(B_{n}-B)X_{n}^{1}I_{G_{n}}||}^{2}|T_{n}\right]\\ & & +(1+\frac{1}{\eta })E[{||(B_{n}-B)\theta I_{G_{n}}-(F_{n}-F)I_{G_{n}}||}^{2}|T_{n}]\\ & \leq & (1+\eta )b{||X_{n}^{1}I_{G_{n}}||}^{2}+2(1+\frac{1}{\eta })(b{||\theta ||}^{2}+d). \end{array}$$

Therefore:

$$\begin{array}{ccc} E[{||X_{n+1}^{1}||}^{2}I_{G_{n+1}}|T_{n}] & \leq & ({\left(1-a\lambda \right)}^{2}+2a(1-a\lambda )\delta +a^{2}(1+\eta )b){||X_{n}^{1}I_{G_{n}}||}^{2}\\ & + & 2a(1-a\lambda )\delta (||\theta ||+1)||X_{n}^{1}I_{G_{n}}||\\ & + & 2a^{2}(1+\frac{1}{\eta })(b{||\theta ||}^{2}+d). \end{array}$$

As $||X_{n}^{1}I_{G_{n}}||\leq 1+{||X_{n}^{1}I_{G_{n}}||}^{2}$, taking mathematical expectation yields:

$$\begin{array}{ccc} E[{||X_{n+1}^{1}||}^{2}I_{G_{n+1}}] & \leq & \rho E[{||X_{n}^{1}I_{G_{n}}||}^{2}]+e,\\ \rho & = & {\left(1-a\lambda \right)}^{2}+2a(1-a\lambda )\delta (||\theta ||+2)+a^{2}(1+\eta )b,\\ e & = & 2a(1-a\lambda )\delta (||\theta ||+1)+2a^{2}(1+\frac{1}{\eta })(b{||\theta ||}^{2}+d). \end{array}$$

As $\rho =1+2a((1-a\lambda )(||\theta ||+2)\delta -\lambda +\frac{a}{2}(\lambda^{2}+b+\eta b))<1$ by the choice of *δ*, this implies $g=\sup_{n}E[{||X_{n}^{1}||}^{2}I_{G_{n}}]<\infty$.

Step 3b: conclusion.

$$\begin{array}{ccc} E[{||(B_{n}-B)X_{n}^{1}I_{G_{n}}||}^{2}] & = & E[E[{||(B_{n}-B)X_{n}^{1}I_{G_{n}}||}^{2}|T_{n}]]\\ & \leq & E[E[{||B_{n}-B||}^{2}|T_{n}]{||X_{n}^{1}I_{G_{n}}||}^{2}]\\ & \leq & bg. \end{array}$$

Then: $\sum_{n=1}^{\infty }\frac{E[{||(B_{n}-B)X_{n}^{1}I_{G_{n}}||}^{2}]}{n^{2}}<\infty$. Therefore a.s.:

$$\begin{array}{c} \frac{1}{n}\sum_{j=1}^{n}((B_{j}-B)X_{j}^{1}I_{G_{j}}-E[(B_{j}-B)X_{j}^{1}I_{G_{j}}|T_{j}])⟶0. \end{array}$$

Now:

$$\begin{array}{c} \sum_{n=1}^{\infty }\frac{1}{n}E[||(E[B_{n}|T_{n}]-B)X_{n}^{1}I_{G_{n}}||]\leq \sum_{n=1}^{\infty }\frac{1}{n}E[||E[B_{n}|T_{n}]-B||||X_{n}^{1}I_{G_{n}}||]\\ \leq \sum_{n=1}^{\infty }\frac{1}{n}{\left(E[{||E[B_{n}|T_{n}]-B||}^{2}]\right)}^{\frac{1}{2}}{\left(E[{||X_{n}^{1}I_{G_{n}}||}^{2}]\right)}^{\frac{1}{2}}\\ \leq g^{\frac{1}{2}}\sum_{n=1}^{\infty }\frac{1}{n}{\left(E[{||E[B_{n}|T_{n}]-B||}^{2}]\right)}^{\frac{1}{2}}<\infty \;\text{by}\;\text{H1b2}. \end{array}$$

Then:

$$\begin{array}{c} \sum_{n=1}^{\infty }\frac{1}{n}||(E[B_{n}|T_{n}]-B)X_{n}^{1}I_{G_{n}}||<\infty \;\text{a}.\text{s}. \end{array}$$

This implies by the Kronecker lemma:

$$\begin{array}{c} \frac{1}{n}\sum_{j=1}^{n}(E[B_{j}|T_{j}]-B)X_{j}^{1}I_{G_{j}}⟶0\;\text{a}.\text{s}. \end{array}$$

Therefore:

$$\begin{array}{c} \frac{1}{n}\sum_{j=1}^{n}(B_{j}-B)X_{j}^{1}I_{G_{j}}⟶0\;\text{a}.\text{s}. \end{array}$$

In *G*, *I*_*G*_*j*__ = 1 for all *j*, therefore $\frac{1}{n}\sum_{j=1}^{n}(B_{j}-B)X_{j}^{1}⟶0$ a.s. Then: $P(\frac{1}{n}\sum_{j=1}^{n}(B_{j}-B)X_{j}^{1}⟶0)\geq P(G)>1-\varepsilon$. This is true for every *ε* > 0. Thus:

$$\begin{array}{c} \frac{1}{n}\sum_{j=1}^{n}(B_{j}-B)X_{j}^{1}⟶0\;\text{a}.\text{s}. \end{array}$$

Therefore by step 2 and step 1, we conclude that $\frac{1}{n}\sum_{j=1}^{n}Z_{j}⟶0$ and $Y_{n}^{1}⟶0$ a.s. ∎

### 3.3 Application to linear regression with online standardized data

Define as in section 2:

$$\begin{array}{ccc} B_{n} & = & \Gamma_{M_{n-1}}\frac{1}{m_{n}}\sum_{j\in I_{n}}(R_{j}-{\bar{R}}_{M_{n-1}}){\left(R_{j}-{\bar{R}}_{M_{n-1}}\right)}^{\prime }\Gamma_{M_{n-1}},\\ F_{n} & = & \Gamma_{M_{n-1}}\frac{1}{m_{n}}\sum_{j\in I_{n}}(R_{j}-{\bar{R}}_{M_{n-1}}){\left(S_{j}-{\bar{S}}_{M_{n-1}}\right)}^{\prime }\Gamma_{M_{n-1}}^{1}. \end{array}$$

Denote *U* = (*R* − *E*[*R*])(*R* − *E*[*R*])′, *B* = Γ*E*[*U*]Γ the correlation matrix of *R*, λ and λ_*max*_ respectively the smallest and the largest eigenvalue of *B*, *b*_1_ = *E*[‖Γ*U*Γ − *B*‖^2^], *F* = Γ*E*[(*R* − *E*[*R*])(*S* − *E*[*S*])′]Γ^1^.

**Corollary 8** *Suppose there is no affine relation between the components of R and the moments of order 4 of* (*R*,*S*) *exist*. *Suppose H3b1 holds*:

(*H3b1*) $0<a<min(\frac{1}{\lambda_{max}},\frac{2\lambda }{\lambda^{2}+b_{1}})$.

*Then Y*_*n*_ *converges to θ* = *B*^−1^*F a.s*.

This process was tested on several datasets and some results are given in section 5 (process S21 for *m*_*n*_ = 1 and S22 for *m*_*n*_ = 10).

*Proof of Corollary 8*.

Step 1: introduction.

Using the decomposition of *E*[*B*_*n*_|*T*_*n*_] − *B* established in the proof of corollary 4, as ${\bar{R}}_{M_{n-1}}⟶E[R]$ and Γ_*M*_*n* − 1__ ⟶ Γ a.s., it is obvious that *E*[*B*_*n*_|*T*_*n*_] − *B* ⟶ 0 a.s. Likewise *E*[*F*_*n*_|*T*_*n*_] − *F* ⟶ 0 a.s. Thus assumptions H1b1 and H2b1 are verified.

Suppose that *Y*_*n*_ does not converge to *θ* almost surely.

Then there exists a set of probability *ε*_1_ > 0 in which *Y*_*n*_ does not converge to *θ*.

Denote $\sigma^{j}=\sqrt{Var[R^{j}]}$, *j* = 1,…,*p*.

As ${\bar{R}}_{M_{n-1}}-E[R]⟶0$, $\sqrt{\frac{M_{n-1}}{M_{n-1}-1}}V_{M_{n-1}}^{j}-\sigma^{j}⟶0$, *j* = 1,…,*p* and Γ_*M*_*n* − 1__ − Γ ⟶ 0 almost surely, there exists a set *G* of probability greater than $1-\frac{\varepsilon_{1}}{2}$ in which these sequences of random variables converge uniformly to *θ*.

Step 2: prove that $\sum_{n=1}^{\infty }\frac{1}{n}{\left(E[||\Gamma_{M_{n-1}}-\Gamma ||I_{G}]\right)}^{\frac{1}{2}}<\infty$.

By step 2 of the proof of lemma 5, we have for *n* > *N*:

$$\begin{array}{ccc} ||\Gamma_{M_{n-1}}-\Gamma ||I_{G} & \leq & \sum_{j=1}^{p}\frac{\left|\frac{M_{n-1}}{M_{n-1}-1}{\left(V_{M_{n-1}}^{j}\right)}^{2}-{\left(\sigma^{j}\right)}^{2}\right|}{\sqrt{\frac{M_{n-1}}{M_{n-1}-1}}V_{M_{n-1}}^{j}\sigma^{j}\left(\sqrt{\frac{M_{n-1}}{M_{n-1}-1}}V_{M_{n-1}}^{j}+\sigma^{j}\right)}I_{G}. \end{array}$$

As in *G*, $\sqrt{\frac{M_{n-1}}{M_{n-1}-1}}V_{M_{n-1}}^{j}$ converges uniformly to *σ*^*j*^ for *j* = 1,…,*p*, there exists *c* > 0 such that

$$\begin{array}{ccc} ||\Gamma_{M_{n-1}}-\Gamma ||I_{G} & \leq & c\sum_{j=1}^{p}|\frac{M_{n-1}}{M_{n-1}-1}{\left(V_{M_{n-1}}^{j}\right)}^{2}-{\left(\sigma^{j}\right)}^{2}|. \end{array}$$

Then there exists *d* > 0 such that

$$\begin{array}{ccc} E[||\Gamma_{M_{n-1}}-\Gamma ||I_{G}] & \leq & \frac{d}{\sqrt{M_{n-1}}}\leq \frac{d}{\sqrt{n-1}}. \end{array}$$

Therefore $\sum_{n=1}^{\infty }\frac{1}{n}{\left(E[||\Gamma_{M_{n-1}}-\Gamma ||I_{G}]\right)}^{\frac{1}{2}}<\infty$.

Step 3: prove that assumption H1b2 is verified in *G*.

Using the decomposition of *E*[*B*_*n*_|*T*_*n*_] − *B* given in step 1 of the proof of corollary 4, with *R*^*c*^ = *R* − *E*[*R*] and ${\bar{R}}_{M_{n-1}}^{c}={\bar{R}}_{M_{n-1}}-E[R]$ yields a.s.:

$$\begin{array}{ccc} (E[B_{n}|T_{n}]-B)I_{G} & = & \left((\Gamma_{M_{n-1}}-\Gamma )E[R^{c}{R^{c}}^{\prime }]\Gamma_{M_{n-1}}+\Gamma E[R^{c}{R^{c}}^{\prime }](\Gamma_{M_{n-1}}-\Gamma \right)\\ & & +\Gamma_{M_{n-1}}{\bar{R}}_{M_{n-1}}^{c}{\left({\bar{R}}_{M_{n-1}}^{c}\right)}^{\prime }\Gamma_{M_{n-1}})I_{G}. \end{array}$$

As in *G*, Γ_*M*_*n*−1__ − Γ and ${\bar{R}}_{M_{n-1}}^{c}$ converge uniformly to 0, *E*[*B*_*n*_|*T*_*n*_] − *B* converges uniformly to 0. Moreover there exists *c*_1_ > 0 such that

$$\begin{array}{ccc} ||E[B_{n}|T_{n}]-B||I_{G} & \leq & c_{1}(||\Gamma_{M_{n-1}}-\Gamma ||I_{G}+||{\bar{R}}_{M_{n-1}}^{c}||)\;\text{a}.\text{s}. \end{array}$$

By the proof of lemma 5: $E[||{\bar{R}}_{M_{n-1}}^{c}||]\leq {\left(\frac{Var[R]}{n-1}\right)}^{\frac{1}{2}}$; then $\sum_{n=1}^{\infty }\frac{1}{n}{\left(E[||{\bar{R}}_{M_{n-1}}^{c}||]\right)}^{\frac{1}{2}}<\infty$.

By step 2: $\sum_{n=1}^{\infty }\frac{1}{n}{\left(E[||\Gamma_{M_{n-1}}-\Gamma ||I_{G}]\right)}^{\frac{1}{2}}<\infty$.

Then: $\sum_{n=1}^{\infty }\frac{1}{n}{\left(E[||E[B_{n}|T_{n}]-B||I_{G}]\right)}^{\frac{1}{2}}<\infty$.

As *E*[*B*_*n*_|*T*_*n*_] − *B* converges uniformly to 0 on *G*, we obtain:

$$\begin{array}{c} \sum_{n=1}^{\infty }\frac{1}{n}{\left(E[{||E[B_{n}|T_{n}]-B||}^{2}I_{G}]\right)}^{\frac{1}{2}}<\infty . \end{array}$$

Thus assumption H1b2 is verified in G.

Step 4: prove that assumption H1b3 is verified in G.

Denote *R*^*c*^ = *R* − *E*[*R*], $R_{j}^{c}=R_{j}-E[R]$, ${\bar{R}}_{j}^{c}={\bar{R}}_{j}-E[R]$. Consider the decomposition:

$$\begin{array}{ccc} B_{n}-B & = & \Gamma_{M_{n-1}}\frac{1}{m_{n}}\sum_{j\in I_{n}}(R_{j}^{c}-{\bar{R}}_{M_{n-1}}^{c}){\left(R_{j}^{c}-{\bar{R}}_{M_{n-1}}^{c}\right)}^{\prime }\Gamma_{M_{n-1}}\\ & & -\Gamma E[R^{c}{R^{c}}^{\prime }]\Gamma \\ & = & \alpha_{n}+\beta_{n} \end{array}$$

$$\begin{array}{ccc} \text{with}\;\alpha_{n} & = & \Gamma_{M_{n-1}}\frac{1}{m_{n}}\sum_{j\in I_{n}}(R_{j}^{c}{R_{j}^{c}}^{\prime }-{\bar{R}}_{M_{n-1}}^{c}{R_{j}^{c}}^{\prime }-R_{j}^{c}{\left({\bar{R}}_{M_{n-1}}^{c}\right)}^{\prime }+{\bar{R}}_{M_{n-1}}^{c}{\left({\bar{R}}_{M_{n-1}}^{c}\right)}^{\prime })\Gamma_{M_{n-1}}\\ & & -\Gamma \frac{1}{m_{n}}\sum_{j\in I_{n}}R_{j}^{c}{R_{j}^{c}}^{\prime }\Gamma \\ & = & \left(\Gamma_{M_{n-1}}-\Gamma )(\frac{1}{m_{n}}\sum_{j\in I_{n}}R_{j}^{c}{R_{j}^{c}}^{\prime })\Gamma_{M_{n-1}}+\Gamma (\frac{1}{m_{n}}\sum_{j\in I_{n}}R_{j}^{c}{R_{j}^{c}}^{\prime })(\Gamma_{M_{n-1}}-\Gamma \right)\\ & & -\Gamma_{M_{n-1}}{\bar{R}}_{M_{n-1}}^{c}\frac{1}{m_{n}}\sum_{j\in I_{n}}{R_{j}^{c}}^{\prime }\Gamma_{M_{n-1}}-\Gamma_{M_{n-1}}\frac{1}{m_{n}}\sum_{j\in I_{n}}R_{j}^{c}{\left({\bar{R}}_{M_{n-1}}^{c}\right)}^{\prime }\Gamma_{M_{n-1}}\\ & & +\Gamma_{M_{n-1}}{\bar{R}}_{M_{n-1}}^{c}{\left({\bar{R}}_{M_{n-1}}^{c}\right)}^{\prime }\Gamma_{M_{n-1}},\\ \beta_{n} & = & \Gamma (\frac{1}{m_{n}}\sum_{j\in I_{n}}R_{j}^{c}{R_{j}^{c}}^{\prime }-E[R^{c}{R^{c}}^{\prime }])\Gamma . \end{array}$$

Let *η* > 0.

$$\begin{array}{ccc} E[{||B_{n}-B||}^{2}I_{G}|T_{n}] & = & E[{||\alpha_{n}+\beta_{n}||}^{2}I_{G}|T_{n}]\\ & \leq & \left(1+\frac{1}{\eta })E[{||\alpha_{n}||}^{2}I_{G}|T_{n}\right]\\ & & +(1+\eta )E[{||\beta_{n}||}^{2}I_{G}|T_{n}]\;\text{a}.\text{s}. \end{array}$$

As random variables $R_{j}^{c}$, *j* ∈ *I*_*n*_, are independent of *T*_*n*_, as Γ_*M*_*n*−1__ and ${\bar{R}}_{M_{n-1}}^{c}$ are *T*_*n*_-measurable and converge uniformly respectively to Γ and 0 on *G*, *E*[‖*α*_*n*_‖^2^ *I*_*G*_|*T*_*n*_] converges uniformly to 0. Then, for *δ* > 0, there exists *N*_1_ such that for *n* > *N*_1_, *E*[‖*α*_*n*_‖^2^ *I*_*G*_|*T*_*n*_] ≤ *δ* a.s.

Moreover, denoting *U* = *R*^*c*^*R*^*c*^′ and $U_{j}=R_{j}^{c}{R_{j}^{c}}^{\prime }$, we have, as the random variables *U*_*j*_ form an i.i.d. sample of *U*:

$$\begin{array}{ccc} E[{||\beta_{n}||}^{2}|T_{n}] & = & E[{||\frac{1}{m_{n}}\sum_{j\in I_{n}}\Gamma (U_{j}-E[U])\Gamma ||}^{2}|T_{n}]\\ & \leq & E[{||\Gamma (U-E[U])\Gamma ||}^{2}]=E[{||\Gamma U\Gamma -E[\Gamma U\Gamma ]||}^{2}]=b_{1}\;\text{a}.\text{s}. \end{array}$$

Then:

$$\begin{array}{ccc} E[{||B_{n}-B||}^{2}I_{G}|T_{n}] & \leq & (1+\frac{1}{\eta })\delta +(1+\eta )b_{1}=b\;\text{a}.\text{s}. \end{array}$$

Thus assumption H1b3 is verified in *G*.

As ${\bar{S}}_{M_{n-1}}-E[S]⟶0$ and $\Gamma_{M_{n-1}}^{1}-\Gamma^{1}⟶0$ almost surely, it can be proved likewise that there exist a set *H* of probability greater than $1-\frac{\varepsilon_{1}}{2}$ and *d* > 0 such that *E*[‖*F*_*n*_ − *F*‖^2^ *I*_*H*_|*T*_*n*_] ≤ *d* a.s. Thus assumption H2b2 is verified in *H*.

Step 5: conclusion.

As $a<min(\frac{1}{\lambda_{max}},\frac{2\lambda }{\lambda^{2}+b_{1}})$, $b_{1}<\frac{2\lambda }{a}-\lambda^{2}$.

Choose $0<\eta <\frac{\frac{2\lambda }{a}-\lambda^{2}}{b_{1}}-1$ and $0<\delta <\frac{\frac{2\lambda }{a}-\lambda^{2}-(1+\eta )b_{1}}{1+\frac{1}{\eta }}$ such that

$$\begin{array}{c} b=(1+\frac{1}{\eta })\delta +(1+\eta )b_{1}<\frac{2\lambda }{a}-\lambda^{2}⟺a<\frac{2\lambda }{\lambda^{2}+b}. \end{array}$$

Thus assumption H3b is verified.

Applying theorem 7 implies that *Y*_*n*_ converges to *θ* almost surely in *H* ∩ *G*.

Therefore *P*(*Y*_*n*_ ⟶ *θ*) ≥ *P*(*H* ∩ *G*) > 1 − *ε*_1_.

This is in contradiction with $P(Y_{n}↛\theta )=\varepsilon_{1}$. Thus *Y*_*n*_ converges to *θ* a.s. ∎

## 4 Convergence of a process with a variable or constant step-size and use of all observations until the current step

In this section, the convergence of the process (*X*_*n*_, *n* ≥ 1) in $R^{p\times q}$ recursively defined by

$$\begin{array}{c} X_{n+1}=X_{n}-a_{n}(B_{n}X_{n}-F_{n}) \end{array}$$

and its application to sequential linear regression are studied.

### 4.1 Theorem

Make the following assumptions

(H1c) There exists a positive definite symmetrical matrix *B* such that *B*_*n*_ ⟶ *B* a.s.

(H2c) There exists a matrix *F* such that *F*_*n*_ ⟶ *F* a.s.

(H3c) λ_*max*_ denoting the largest eigenvalue of *B*,

$\left(a_{n}=a<\frac{1}{\lambda_{max}}\right)$ or $\left(a_{n}⟶0,\sum_{n=1}^{\infty }a_{n}=\infty \right)$.

**Theorem 9** *Suppose H1c, H2c and H3c hold*. *Then X*_*n*_ *converges to B*^−1^*F a.s*.

*Proof of Theorem 9*.

Denote *θ* = *B*^−1^*F*, $X_{n}^{1}=X_{n}-\theta$, *Z*_*n*_ = (*B*_*n*_ − *B*)*θ* − (*F*_*n*_ − *F*). Then:

$$\begin{array}{c} X_{n+1}^{1}=(I-a_{n}B_{n})X_{n}^{1}-a_{n}Z_{n}. \end{array}$$

Let *ω* be fixed belonging to the intersection of the convergence sets {*B*_*n*_ ⟶ *B*} and {*F*_*n*_ ⟶ *F*}. The writing of *ω* is omitted in the following.

Denote ‖*A*‖ the spectral norm of a matrix *A* and λ the smallest eigenvalue of *B*.

In the case *a*_*n*_ depending on *n*, as *a*_*n*_ ⟶ 0, we can suppose without loss of generality $a_{n}<\frac{1}{\lambda_{max}}$ for all *n*. Then all the eigenvalues of *I* − *a*_*n*_*B* are positive and ‖*I* − *a*_*n*_*B*‖ = 1 − *a*_*n*_λ.

Let 0 < *ε* < λ. As *B*_*n*_ − *B* ⟶ 0, we obtain for *n* sufficiently large:

$$\begin{array}{ccc} ||I-a_{n}B_{n}|| & \leq & ||I-a_{n}B||+a_{n}||B_{n}-B||\\ & \leq & 1-a_{n}\lambda +a_{n}\varepsilon \;\text{,}\;\text{with}\;a_{n}<\frac{1}{\lambda -\varepsilon }\\ ||X_{n+1}^{1}|| & \leq & (1-a_{n}(\lambda -\varepsilon ))||X_{n}^{1}||+a_{n}||Z_{n}||. \end{array}$$

As *Z*_*n*_ ⟶ 0, applying lemma 6 yields $||X_{n}^{1}||⟶0$.

Therefore *X*_*n*_ ⟶ *B*^−1^*F* a.s. ∎

### 4.2 Application to linear regression with online standardized data

Let (*m*_*n*_, *n* ≥ 1) be a sequence of integers. Denote $M_{n}=\sum_{k=1}^{n}m_{k}$ for *n* ≥ 1, *M*_0_ = 0 and *I*_*n*_ = {*M*_*n* − 1_ + 1,…,*M*_*n*_}.

Define

$$\begin{array}{ccc} B_{n} & = & \Gamma_{M_{n}}(\frac{1}{M_{n}}\sum_{i=1}^{n}\sum_{j\in I_{i}}R_{j}R_{j}^{\prime }-{\bar{R}}_{M_{n}}{\bar{R}}_{M_{n}}^{\prime })\Gamma_{M_{n}},\\ F_{n} & = & \Gamma_{M_{n}}(\frac{1}{M_{n}}\sum_{i=1}^{n}\sum_{j\in I_{i}}R_{j}S_{j}^{\prime }-{\bar{R}}_{M_{n}}{\bar{S}}_{M_{n}}^{\prime })\Gamma_{M_{n}}^{1}. \end{array}$$

As ((*R*_*n*_, *S*_*n*_), *n* ≥ 1) is an i.i.d. sample of (*R*, *S*), assumptions H1c and H2c are obviously verified with *B* = Γ*E*[(*R* − *E*[*R*])(*R* − *E*[*R*])′]Γ and *F* = Γ*E*[(*R* − *E*[*R*])(*S* − *E*[*S*])′]Γ^1^. Then:

**Corollary 10** *Suppose there is no affine relation between the components of R and the moments of order 4 of* (*R*, *S*) *exist*. *Suppose H3c holds*. *Then X*_*n*_ *converges to B*^−1^*F a.s*.

**Remark 3** *B is the correlation matrix of R of dimension p*. *Then λ*_*max*_ < *Trace*(*B*) = *p*. *In the case of a constant step-size a*, *it suffices to take* $a\leq \frac{1}{p}$ *to verify H3c*.

**Remark 4** *In the definition of B*_*n*_ *and F*_*n*_, *the R*_*j*_ *and the S*_*j*_ *are not directly pseudo-centered with respect to* ${\bar{R}}_{M_{n}}$ *and* ${\bar{S}}_{M_{n}}$ *respectively*. *Another equivalent definition of B*_*n*_ *and F*_*n*_ *can be used*. *It consists of replacing R*_*j*_ *by R*_*j*_ − *m*, ${\bar{R}}_{M_{n}}$ *by* ${\bar{R}}_{M_{n}}-m$, *S*_*j*_ *by S*_*j*_ − *m*, ${\bar{S}}_{M_{n}}$ *by* ${\bar{S}}_{M_{n}}-m_{1}$, *m and m*_1_ *being respectively an estimation of E*[*R*] *and E*[*S*] *computed in a preliminary phase with a small number of observations*. *For example, at step n*, $\sum_{j\in I_{n}}\Gamma_{M_{n}}\left(R_{j}-m\right){\left(\Gamma_{M_{n}}\left(R_{j}-m\right)\right)}^{\prime }$ *is computed instead of* $\sum_{j\in I_{n}}\Gamma_{M_{n}}R_{j}{\left(\Gamma_{M_{n}}R_{j}\right)}^{\prime }$. *This limits the risk of numerical explosion*.

This process was tested on several datasets and some results are given in section 5 (with a variable step-size: process S13 for *m*_*n*_ = 1 and S14 for *m*_*n*_ = 10; with a constant step-size: process S31 for *m*_*n*_ = 1 and S32 for *m*_*n*_ = 10).

## 5 Experiments

The three previously-defined processes of stochastic approximation with online standardized data were compared with the classical stochastic approximation and averaged stochastic approximation (or averaged stochastic gradient descent) processes with constant step-size (denoted ASGD) studied in [5] and [6]. A description of the methods along with abbreviations and parameters used is given in Table 1.

**Table 1:** Description of the methods.

Method type	Abbreviation	Type of data	Number of observations used at each step of the process	Use of all the observations until the current step	Step-size	Use of the averaged process
**Classic**	C1	Raw data	1	No	variable	No
	C2		10			
	C3		1	Yes		
	C4		10			
**ASGD**	A1		1	No	constant	Yes
	A2		1			
**Standardization 1**	S11	Online standardized data	1	No	variable	No
	S12		10			
	S13		1	Yes		
	S14		10			
**Standardization 2**	S21		1	No	constant	Yes
	S22		10			
**Standardization 3**	S31		1	Yes		No
	S32		10			

With the variable *S* set at dimension 1, 11 datasets were considered, some of which are available in free access on the Internet, while others were derived from the EPHESUS study [15]: 6 in regression (continuous dependent variable) and 5 in linear discriminant analysis (binary dependent variable). All datasets used in our experiments are presented in detail in Table 2, along with their download links. An *a priori* selection of variables was performed on each dataset using a stepwise procedure based on Fisher’s test with p-to-enter and p-to-remove fixed at 5 percent.

**Table 2:** Datasets used in our experiments.

Dataset name	*N*	*p*_*a*_	*p*	Type of dependent variable	*T*^2^	Number of outliers	
**CADATA**	20640	8	8	Continuous	1.6x10^6^	122	www.dcc.fc.up.pt/∼ltorgo/Regression/DataSets.html
**AILERONS**	7154	40	9	Continuous	247.1	0	www.dcc.fc.up.pt/∼ltorgo/Regression/DataSets.html
**ELEVATORS**	8752	18	10	Continuous	7.7x10^4^	0	www.dcc.fc.up.pt/∼ltorgo/Regression/DataSets.html
**POLY**	5000	48	12	Continuous	4.1x10^4^	0	www.dcc.fc.up.pt/∼ltorgo/Regression/DataSets.html
**eGFR**	21382	31	15	Continuous	2.9x10^4^	0	derived from EPHESUS study [15]
**HEMG**	21382	31	17	Continuous	6.0x10^4^	0	derived from EPHESUS study [15]
**QUANTUM**	50000	78	14	Binary	22.5	1068	www.osmot.cs.cornell.edu/kddcup
**ADULT**	45222	97	95	Binary	4.7x10^10^	20	www.cs.toronto.edu/∼delve/data/datasets.html
**RINGNORM**	7400	20	20	Binary	52.8	0	www.cs.toronto.edu/∼delve/data/datasets.html
**TWONORM**	7400	20	20	Binary	24.9	0	www.cs.toronto.edu/∼delve/data/datasets.html
**HOSPHF30D**	21382	32	15	Binary	8.1x10^5^	0	derived from EPHESUS study [15]

*N* denotes the size of global sample, *p*_*a*_ the number of parameters available, *p* the number of parameters selected and *T*^2^ the trace of *E*[*RR*′]. Outlier is defined as an observation whose the L2 norm is greater than five times the average norm.

Let *D* = {(*r*_*i*_, *s*_*i*_), *i* = 1, 2,…,*N*} be the set of data in $R^{p}\times R$ and assuming that it represents the set of realizations of a random vector (*R*, *S*) uniformly distributed in *D*, then minimizing *E*[(*S* − *θ*′ *R* − *η*)^2^] is equivalent to minimizing $\frac{1}{N}\sum_{i=1}^{N}{\left(s_{i}-\theta^{\prime }r_{i}-\eta \right)}^{2}$. One element of *D* (or several according to the process) is randomly drawn at each step to iterate the process.

To compare the methods, two different studies were performed: one by setting the total number of observations used, the other by setting the computing time.

The choice of step-size, the initialization for each method and the convergence criterion used are respectively presented and commented below.

**Choice** **of step-size**

In all methods of stochastic approximation, a suitable choice of step-size is often crucial for obtaining good performance of the process. If the step-size is too small, the convergence rate will be slower. Conversely, if the step-size is too large, a numerical explosion phenomenon may occur during the first iterations.

For the processes with a variable step-size (processes C1 to C4 and S11 to S14), we chose to use *a*_*n*_ of the following type:

$$\begin{array}{ccc} a_{n} & = & \frac{c_{\gamma }}{{\left(b+n\right)}^{\alpha }}. \end{array}$$

The constant $\alpha =\frac{2}{3}$ was fixed, as suggested by Xu [16] in the case of stochastic approximation in linear regression, and *b* = 1. The results obtained for the choice $c_{\gamma }=\frac{1}{p}$ are presented although the latter does not correspond to the best choice for a classical method.

For the ASGD method (A1, A2), two different constant step-sizes *a* as used in [6] were tested: $a=\frac{1}{T^{2}}$ and $a=\frac{1}{2T^{2}}$, *T*^2^ denoting the trace of *E*[*RR*′]. Note that this choice of constant step-size assumes knowing *a priori* the dataset and is not suitable for a data stream.

For the methods with standardization and a constant step-size *a* (S21, S22, S31, S32), $a=\frac{1}{p}$ was chosen since the matrix *E*[*RR*′] is thus the correlation matrix of *R*, whose trace is equal to *p*, such that this choice corresponds to that of [6].

**Initialization** **of processes**

All processes (*X*_*n*_) were initialized by $X_{1}=\underset{-}{0}$, the null vector. For the processes with standardization, a small number of observations (*n* = 1000) were taken into account in order to calculate an initial estimate of the means and standard deviations.

**Convergence** **criterion**

The “theoretical vector” *θ*^1^ is assigned as that obtained by the least square method in *D* such that $\theta^{1^{\prime }}=(\begin{array}{cc} \theta^{\prime } & \eta \end{array})$. Let $\Theta_{n+1}^{1}$ be the estimator of *θ*^1^ obtained by stochastic approximation after *n* iterations.

In the case of a process (*X*_*n*_) with standardized data, which yields an estimation of the vector denoted *θ*_*c*_ in section 1 as *θ* = Γ*θ*_*c*_(Γ^1^)^−1^ and *η* = *E*[*S*] − *θ*′ *E*[*R*], we can define:

$$\begin{array}{ccc} \Theta_{n+1}^{1}{}^{\prime } & = & \left(\begin{array}{cc} \Theta_{n+1}^{\prime } & H_{n+1} \end{array}\right)\\ \text{with}\;\Theta_{n+1} & = & \Gamma_{M_{n}}X_{n+1}{\left(\Gamma_{M_{n}}^{1}\right)}^{-1}\\ H_{n+1} & = & {\bar{S}}_{M_{n}}-\Theta_{n+1}^{\prime }{\bar{R}}_{M_{n}}. \end{array}$$

To judge the convergence of the method, the cosine of the angle formed by exact *θ*^1^ and its estimation $\theta_{n+1}^{1}$ was used as criterion,

$$\begin{array}{c} \text{cos}(\theta^{1},\theta_{n+1}^{1})=\frac{{\theta^{1}}^{\prime }\theta_{n+1}^{1}}{{||\theta^{1}||}_{2}\;{||\theta_{n+1}^{1}||}_{2}}. \end{array}$$

Other criteria, such as $\frac{{||\theta^{1}-\theta_{n+1}^{1}||}_{2}}{{||\theta^{1}||}_{2}}$ or $\frac{f\left(\theta_{n+1}^{1}\right)-f\left(\theta^{1}\right)}{f(\theta^{1})}$, *f* being the loss function, were also tested, although the results are not presented in this article.

### 5.1 Study for a fixed total number of observations used

For all *N* observations used by the algorithm (*N* being the size of *D*) up to a maximum of 100*N* observations, the criterion value associated with each method and for each dataset was recorded. The results obtained after using 10*N* observations are provided in Table 3.

**Table 3:** Results after using 10N observations.

	CADATA	AILERONS	ELEVATORS	POLY	EGFR	HEMG	QUANTUM	ADULT	RINGNORM	TWONORM	HOSPHF30D	Mean rank
**C1**	Expl.	-0.0385	Expl.	Expl.	Expl.	Expl.	0.9252	Expl.	0.9998	1.0000	Expl.	11.6
**C2**	Expl.	0.0680	Expl.	Expl.	Expl.	Expl.	0.8551	Expl.	0.9976	0.9996	Expl.	12.2
**C3**	Expl.	0.0223	Expl.	Expl.	Expl.	Expl.	0.9262	Expl.	0.9999	1.0000	Expl.	9.9
**C4**	Expl.	-0.0100	Expl.	Expl.	Expl.	Expl.	0.8575	Expl.	0.9981	0.9996	Expl.	12.3
**A1**	-0.0013	0.4174	0.0005	0.3361	0.2786	0.2005	Expl.	0.0027	0.9998	1.0000	0.0264	9.2
**A2**	0.0039	0.2526	0.0004	0.1875	0.2375	0.1846	0.0000	0.0022	0.9999	1.0000	0.2047	8.8
**S11**	1.0000	0.9516	0.9298	1.0000	1.0000	0.9996	0.9999	0.7599	0.9999	1.0000	0.7723	5.2
**S12**	0.9999	0.9579	0.9311	1.0000	0.9999	0.9994	0.9991	0.6842	0.9999	1.0000	0.4566	6.1
**S13**	1.0000	0.9802	0.9306	1.0000	1.0000	0.9998	1.0000	0.7142	0.9999	1.0000	0.7754	3.7
**S14**	0.9999	0.9732	0.9303	1.0000	0.9999	0.9994	0.9991	0.6225	0.9998	1.0000	0.4551	6.9
**S21**	0.9993	0.6261	0.9935	Expl.	Expl.	Expl.	Expl.	Expl.	0.9998	1.0000	Expl.	10.5
**S22**	1.0000	0.9977	0.9900	1.0000	1.0000	0.9989	0.9999	-0.0094	0.9999	1.0000	0.9454	4.1
**S31**	1.0000	0.9988	0.9999	1.0000	1.0000	0.9992	0.9999	0.9907	0.9999	1.0000	0.9788	2.3
**S32**	1.0000	0.9991	0.9998	1.0000	1.0000	0.9992	0.9999	0.9867	0.9999	1.0000	0.9806	2.2

Expl. means numerical explosion.

**Fig 1:**
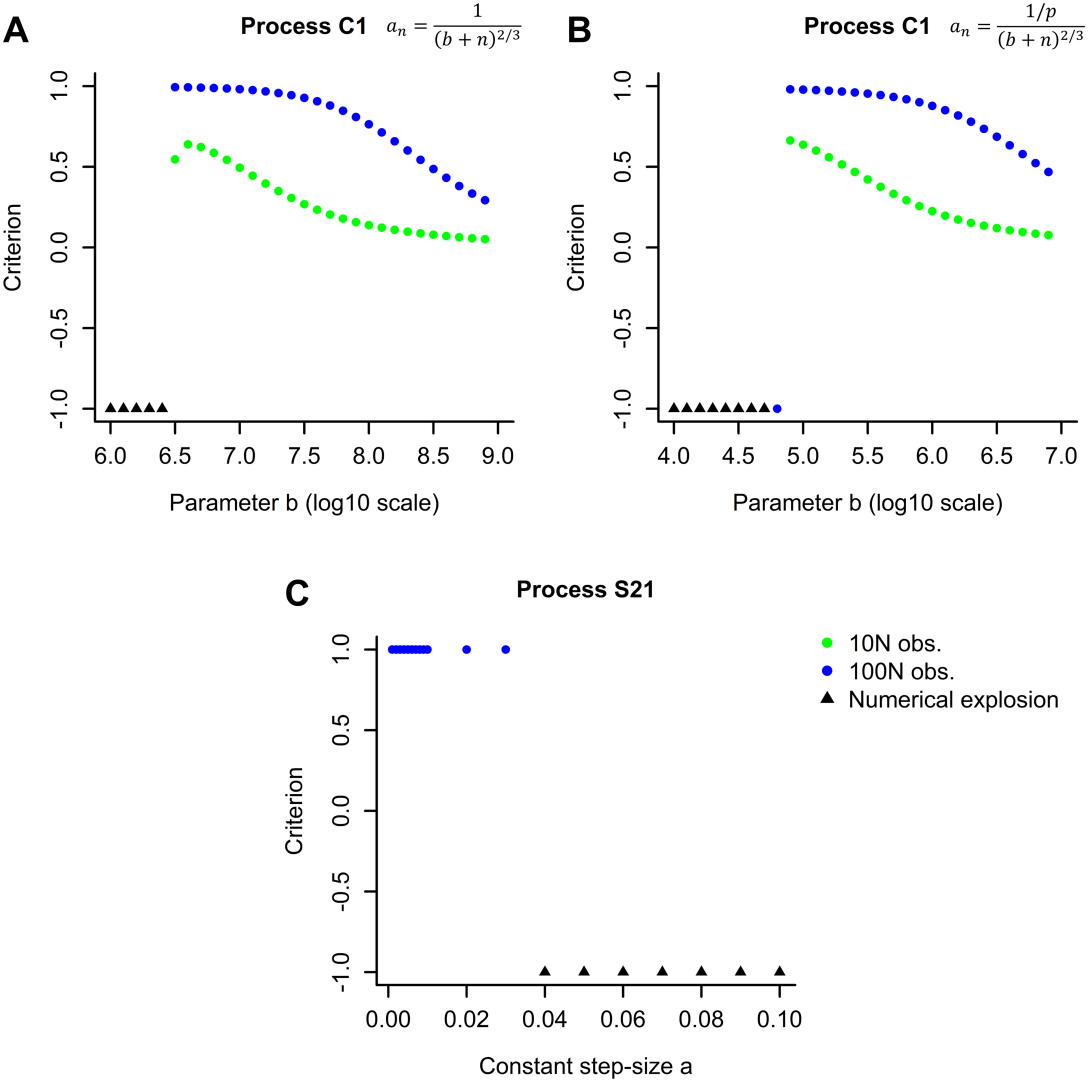
Results obtained for dataset POLY using 10*N* and 100*N* observations: A/ process C1 with variable step-size $a_{n}=\frac{1}{{\left(b+n\right)}^{\frac{2}{3}}}$ by varying *b*, B/ process C1 with variable step-size $a_{n}=\frac{\frac{1}{p}}{{\left(b+n\right)}^{\frac{2}{3}}}$ by varying *b*, C/ process S21 by varying constant step-size *a*.

As can be seen in Table 3, a numerical explosion occured in most datasets using the classical methods with raw data and a variable step-size (C1 to C4). As noted in Table 2, these datasets had a high *T*^2^ = *Tr*(*E*[*RR*′]). Corresponding methods S11 to S14 using the same variable step-size but with online standardized data quickly converged in most cases. However classical methods with raw data can yield good results for a suitable choice of step-size, as demonstrated by the results obtained for POLY dataset in Fig 1. The numerical explosion can arise from a too high step-size when *n* is small. This phenomenon can be avoided if the step-size is reduced, although if the latter is too small, the convergence rate will be slowed. Hence, the right balance must be found between step-size and convergence rate. Furthermore, the choice of this step-size generally depends on the dataset which is not known *a priori* in the case of a data stream. In conclusion, methods with standardized data appear to be more robust to the choice of step-size.

The ASGD method (A1 with constant step-size $a=\frac{1}{T^{2}}$ and A2 with $a=\frac{1}{2T^{2}}$) did not yield good results except for the RINGNORM and TWONORM datasets which were obtained by simulation (note that all methods functioned very well for these two datasets). Of note, A1 exploded for the QUANTUM dataset containing 1068 observations (2.1%) whose L2 norm was fivefold greater than the average norm (Table 2). The corresponding method S21 with online standardized data yielded several numerical explosions with the $a=\frac{1}{p}$ step-size, however these explosions disappeared when using a smaller step-size (see Fig 1). Of note, it is assumed in corollary 8 that $0<a<\min(\frac{1}{\lambda_{max}},\frac{2\lambda }{\lambda^{2}+b_{1}})$; in the case of $a=\frac{1}{p}$, only $a<\frac{1}{\lambda_{max}}$ is certain.

Finally, for methods S31 and S32 with standardized data, the use of all observations until the current step and the very simple choice of the constant step-size $a=\frac{1}{p}$ uniformly yielded good results.

**Fig 2:**
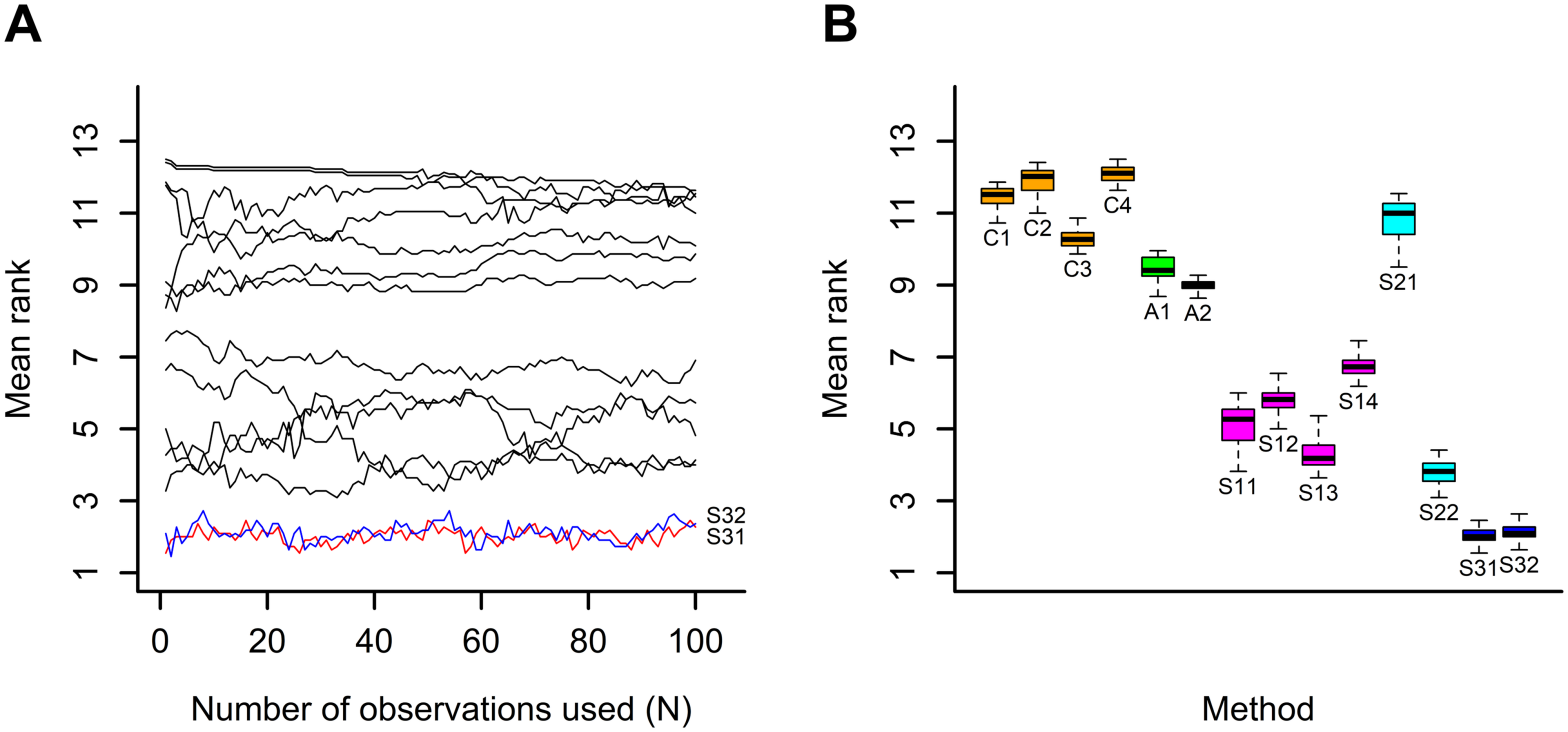
Results for a fixed total number of observations used: A/ change in the mean rank based on the number of observations used, B/ boxplot of the mean rank by method.

Thereafter, for each fixed number of observations used and for each dataset, the 14 methods ranging from the best (the highest cosine) to the worst (the lowest cosine) were ranked by assigning each of the latter a rank from 1 to 14 respectively, after which the mean rank in all 11 datasets was calculated for each method. A total of 100 mean rank values were calculated for a number of observations used varying from *N* to 100*N*. The graph depicting the change in mean rank based on the number of observations used and the boxplot of the mean rank are shown in Fig 2.

Overall, for these 11 datasets, a method with standardized data, a constant step-size and use of all observations until the current step (S31, S32) represented the best method when the total number of observations used was fixed.

### 5.2 Study for a fixed processing time

For every second up to a maximum of 2 minutes, the criterion value associated to each dataset was recorded. The results obtained after a processing time of 1 minute are provided in Table 4.

**Table 4:** Results obtained after a fixed time of 1 minute.

	CADATA	AILERONS	ELEVATORS	POLY	EGFR	HEMG	QUANTUM	ADULT	RINGNORM	TWONORM	HOSPHF30D	Mean rank
**C1**	Expl.	-0.2486	Expl.	Expl.	Expl.	Expl.	0.9561	Expl.	1.0000	1.0000	Expl.	12.2
**C2**	Expl.	0.7719	Expl.	Expl.	Expl.	Expl.	0.9519	Expl.	1.0000	1.0000	Expl.	9.9
**C3**	Expl.	0.4206	Expl.	Expl.	Expl.	Expl.	0.9547	Expl.	1.0000	1.0000	Expl.	10.6
**C4**	Expl.	0.0504	Expl.	Expl.	Expl.	Expl.	0.9439	Expl.	1.0000	1.0000	Expl.	10.1
**A1**	-0.0067	0.8323	0.0022	0.9974	0.7049	0.2964	Expl.	0.0036	1.0000	1.0000	Expl.	9.0
**A2**	0.0131	0.8269	0.0015	0.9893	0.5100	0.2648	Expl.	0.0027	1.0000	1.0000	0.2521	8.6
**S11**	1.0000	0.9858	0.9305	1.0000	1.0000	1.0000	1.0000	0.6786	1.0000	1.0000	0.9686	5.8
**S12**	1.0000	0.9767	0.9276	1.0000	1.0000	0.9999	1.0000	0.6644	1.0000	1.0000	0.9112	5.8
**S13**	1.0000	0.9814	0.9299	1.0000	1.0000	0.9999	1.0000	0.4538	1.0000	1.0000	0.9329	6.1
**S14**	1.0000	0.9760	0.9274	1.0000	1.0000	1.0000	0.9999	0.5932	1.0000	1.0000	0.8801	6.1
**S21**	-0.9998	0.2424	0.6665	Expl.	Expl.	Expl.	Expl.	0.0000	1.0000	1.0000	Expl.	11.5
**S22**	1.0000	0.9999	1.0000	1.0000	1.0000	1.0000	1.0000	-0.0159	1.0000	1.0000	0.9995	3.1
**S31**	1.0000	0.9995	1.0000	1.0000	1.0000	0.9999	1.0000	0.9533	1.0000	1.0000	0.9997	4.5
**S32**	1.0000	0.9999	1.0000	1.0000	1.0000	1.0000	1.0000	0.9820	1.0000	1.0000	0.9999	1.5

Expl. means numerical explosion.

The same conclusions can be drawn as those described in section 5.1 for the classical methods and the ASGD method. The methods with online standardized data typically faired better.

**Fig 3:**
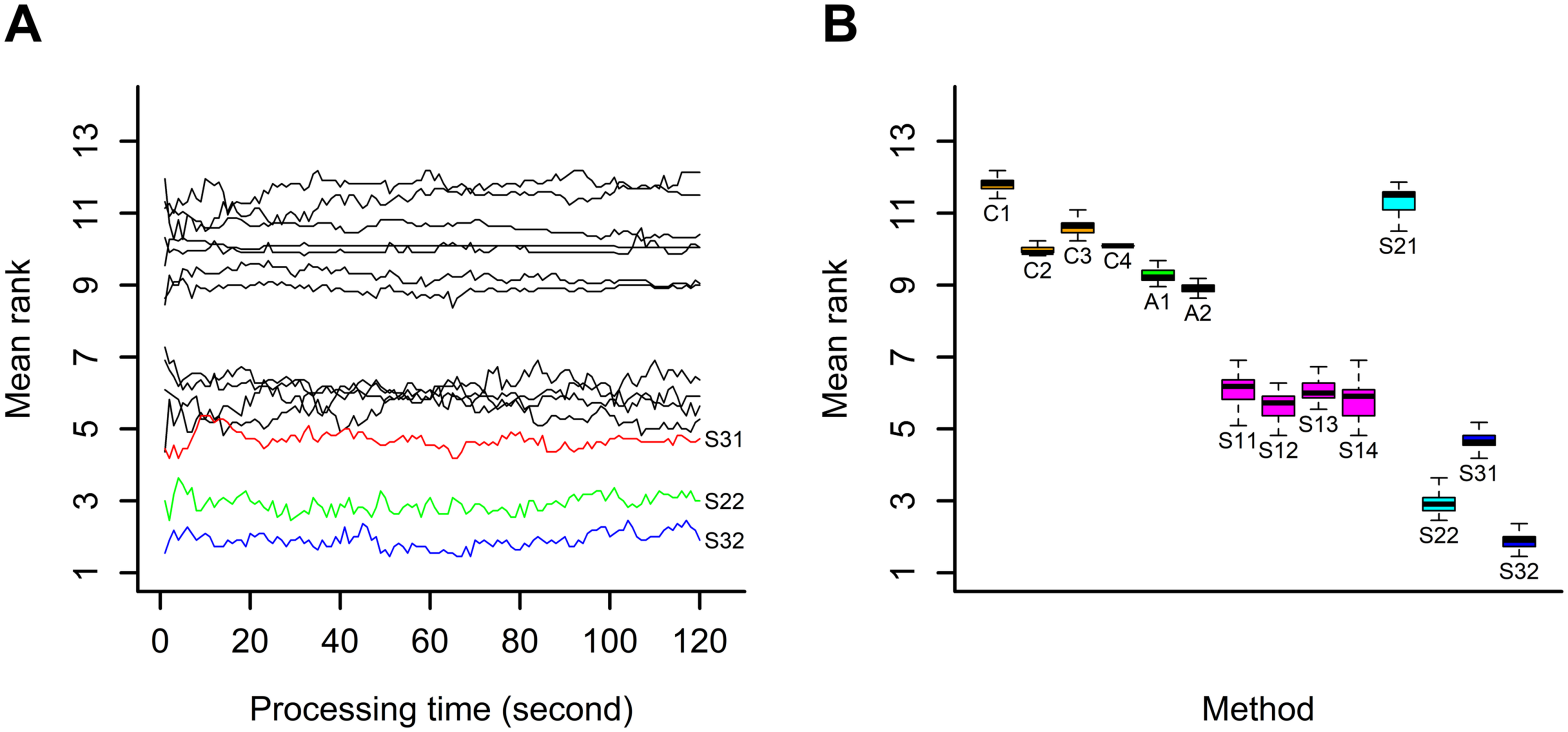
Results for a fixed processing time: A/ change in the mean rank based on the processing time, B/ boxplot of the mean rank by method.

As in the previous study in section 5.1, the 14 methods were ranked from the best to the worst on the basis of the mean rank for a fixed processing time. The graph depicting the change in mean rank based on the processing time varying from 1 second to 2 minutes as well as the boxplot of the mean rank are shown in Fig 3.

As can be seen, these methods with online standardized data using more than one observation per step yielded the best results (S32, S22). One explanation may be that the total number of observations used in a fixed processing time is higher when several observations are used per step rather than one observation per step. This can be verified in Table 5 in which the total number of observations used per second for each method and for each dataset during a processing time of 2 minutes is given. Of note, the number of observations used per second in a process with standardized data and one observation per step (S11, S13, S21, S31) was found to be generally lower than in a process with raw data and one observation per step (C1, C3, A1, A2), since a method with standardization requires the recursive estimation of means and variances at each step.

**Table 5:** Number of observations used after 2 minutes (expressed in number of observations per second).

	CADATA	AILERONS	ELEVATORS	POLY	EGFR	HEMG	QUANTUM	ADULT	RINGNORM	TWONORM	HOSPHF30D
**C1**	19843	33170	17133	14300	10979	9243	33021	476	31843	31677	10922
**C2**	166473	291558	159134	134249	104152	89485	281384	4565	262847	261881	102563
**C3**	17206	28985	16036	13449	10383	8878	28707	462	28123	28472	10404
**C4**	132088	194031	125880	106259	87844	76128	184386	4252	171711	166878	86895
**A1**	33622	35388	36540	35800	35280	34494	11815	15390	34898	34216	14049
**A2**	33317	32807	36271	35628	35314	34454	15439	16349	34401	34205	34890
**S11**	17174	17133	17166	16783	15648	14764	16296	1122	14067	13836	14334
**S12**	45717	47209	45893	43470	39937	37376	40943	4554	34799	34507	36389
**S13**	12062	12731	11888	12057	11211	10369	11466	620	9687	9526	10137
**S14**	43674	46080	43068	42123	38350	35338	39170	4512	33594	31333	32701
**S21**	15396	17997	16772	10265	8404	7238	9166	996	13942	13274	7672
**S22**	47156	47865	46318	43899	40325	37467	41320	4577	34478	31758	37418
**S31**	12495	12859	12775	12350	11495	10619	11608	621	9890	9694	10863
**S32**	44827	47035	45123	42398	38932	36288	39362	4532	33435	33385	35556

Of note, for the ADULT dataset with a large number of parameters selected (95), the only method yielding sufficiently adequate results after a processing time of one minute was S32, and methods S31 and S32 when 10*N* observations were used.

## 6 Conclusion

In the present study, three processes with online standardized data were defined and for which their a.s. convergence was proven.

A stochastic approximation method with standardized data appears to be advantageous compared to a method with raw data. First, it is easier to choose the step-size. For processes S31 and S32 for example, the definition of a constant step-size only requires knowing the number of parameters *p*. Secondly, the standardization usually allows avoiding the phenomenon of numerical explosion often obtained in the examples given with a classical method.

The use of all observations until the current step can reduce the influence of outliers and increase the convergence rate of a process. Moreover, this approach is particularly adapted to the case of a data stream.

Finally, among all processes tested on 11 different datasets (linear regression or linear discriminant analysis), the best was a method using standardization, a constant step-size equal to $\frac{1}{p}$ and all observations until the current step, and the use of several new observations at each step improved the convergence rate.
